# Supplementary material for: Optical N-invariant of graphene’s topological viscous Hall fluid
Source: Nat Commun. 2021 Aug 5;12:4729. doi: 10.1038/s41467-021-25097-2 (PMC8342470; doi:10.1038/s41467-021-25097-2)
Supplement: Supplementary file 1 — Supplementary Information [file 41467_2021_25097_MOESM1_ESM.pdf]

Supplementary Information for "Optical N-invariant of graphene's topological viscous Hall fluid"

Todd Van Mechelen,[1] Wenbo Sun,[1] and Zubin Jacob[1,*]

[1]*Purdue University, School of Electrical and Computer Engineering,*
Brick Nanotechnology Center, 47907, West Lafayette, Indiana, USA

Here we present the detailed derivations of the optical N-invariant and Fresnel equations for a two-dimensional parity and time-reversal breaking electron fluid. The Fresnel equations represent the scattering theory from a surface charge density. We also derive the dynamical variables of the bound modes (bulk magnetoplasmons) which correspond to poles of the reflection matrix. We conclude with a rigorous analysis of the boundary edge physics and demonstrate the robustness of the gapless edge plasmons. To avoid confusion, we denote three-dimensional (3D) vectors with arrows $\vec{\mathcal{A}} = (\mathcal{A}_x, \mathcal{A}_y, \mathcal{A}_z)$ and reserve boldface for two-dimensional (2D) vectors $\mathbf{A} = (A_x, A_y)$ which corresponds to strictly in-plane fields.

Contents

Supplementary Note 1: Boundary conditions of a semi-infinite interface with surface charge 2
2D linear response theory 3

Supplementary Note 2: Navier-Stokes equations of the viscous Hall fluid 3
Magnetohydrodynamic linear response theory 4

Supplementary Note 3: Optical N-invariant 6
Homotopy equivalence of susceptibility and conductivity 7
Topological quantization and effect of dissipation 7
Optical N-invariant of the viscous Hall fluid 8

Supplementary Note 4: Fresnel equations of 2D electron fluids 10

Supplementary Note 5: Viscous bulk magnetoplasmons 12
Bulk dispersion relation 13
Dynamical variables of the viscous Hall fluid: energy and angular momentum 13

Supplementary Note 6: Topological edge magnetoplasmons 16
Fetter approximation 17
Boundary conditions and characteristic equation 18
Ultra-subwavelength topological circulators 20

Supplementary References 20

*Electronic address: zjacob@purdue.edu

Supplementary Note 1: Boundary conditions of a semi-infinite interface with surface charge

We consider a semi-infinite interface in the z direction and unbounded in the x-y plane. In this case, we have a two-dimensional (2D) material located at $z = 0$, which amounts to a free surface charge, and arbitrary three-dimensional (3D) materials in the $z > 0$ and $z < 0$ spaces. To include the the 2D material in a 3D theory, we let the surface charges at the interface be described as,

$$\vec{\mathcal{J}}(t, \vec{r}) = \delta(z)\mathbf{J}(t, \mathbf{r}), \qquad \rho(t, \vec{r}) = \delta(z)\rho(t, \mathbf{r}), \tag{1}$$

where $\delta(z)$ is the Dirac delta function localized at $z = 0$ and $\mathbf{J} = (J_x, J_y)$ is a 2D current. This current is conserved in the $\mathbf{r} = (x, y)$ plane and obeys the 2D continuity equation,

$$\frac{\partial}{\partial t}\rho(t, \mathbf{r}) + \boldsymbol{\nabla} \cdot \mathbf{J}(t, \mathbf{r}) = 0. \tag{2}$$

The differential form of Maxwell's equations are then expressed in Gaussian units as,

$$\vec{\nabla} \cdot \vec{\mathcal{D}}(t, \vec{r}) = 4\pi\rho(t, \vec{r}) = 4\pi\delta(z)\rho(t, \mathbf{r}), \tag{3a}$$

$$\vec{\nabla} \times \vec{\mathcal{H}}(t, \vec{r}) = \frac{4\pi}{c}\vec{\mathcal{J}}(t, \vec{r}) + \frac{1}{c}\frac{\partial}{\partial t}\vec{\mathcal{D}}(t, \vec{r}) = \frac{4\pi}{c}\delta(z)\mathbf{J}(t, \mathbf{r}) + \frac{1}{c}\frac{\partial}{\partial t}\vec{\mathcal{D}}(t, \vec{r}), \tag{3b}$$

where $\vec{\mathcal{D}}$ and $\vec{\mathcal{H}}$ are the electric displacement and auxiliary magnetic fields respectively. $\vec{\mathcal{D}}$ and $\vec{\mathcal{H}}$ capture the field + material response in the 3D half-spaces $z > 0$ and $z < 0$ while the free surface charge $\mathbf{J}$ represents the response of the 2D material at $z = 0$. The remaining equations relate the electric field $\vec{\mathcal{E}}$ to the magnetic field $\vec{\mathcal{B}}$,

$$\vec{\nabla} \cdot \vec{\mathcal{B}}(t, \vec{r}) = 0, \tag{3c}$$

$$\vec{\nabla} \times \vec{\mathcal{E}}(t, \vec{r}) = -\frac{1}{c}\frac{\partial}{\partial t}\vec{\mathcal{B}}(t, \vec{r}). \tag{3d}$$

The boundary conditions are well known and can be derived from the integral forms of Maxwell's equations [1]. We repeat them here for completeness. Integrating Supplementary Eq. (3a) over an infinitesimal width along z, the boundary condition on the displacement field $\vec{\mathcal{D}}$ reads,

$$\boxed{\mathcal{D}_{z,+}(t, \mathbf{r}, 0^+) - \mathcal{D}_{z,-}(t, \mathbf{r}, 0^-) = \Delta\mathcal{D}_z(t, \mathbf{r}) = 4\pi\rho(t, \mathbf{r})}, \tag{4a}$$

where the labels $+$ and $-$ correspond to the fields immediately in the $z > 0$ and $z < 0$ spaces respectively. Repeating in a similar manner for Supplementary Eq. (3b), the boundary condition on the auxiliary field $\vec{\mathcal{H}}$ is,

$$\boxed{\mathcal{H}_{i,+}(t, \mathbf{r}, 0^+) - \mathcal{H}_{i,-}(t, \mathbf{r}, 0^-) = \Delta\mathcal{H}_i(t, \mathbf{r}) = \frac{4\pi}{c}\epsilon_{ij}J^j(t, \mathbf{r})}, \tag{4b}$$

where $i = x, y$ labels in-plane indices and $\epsilon_{ij} = -\epsilon_{ji}$ is the antisymmetric matrix, which should not be confused with permittivity. The boundary conditions on the electric $\vec{\mathcal{E}}$ and $\vec{\mathcal{B}}$ magnetic fields are simply,

$$\boxed{\mathcal{E}_{i,+}(t, \mathbf{r}, 0^+) = \mathcal{E}_{i,-}(t, \mathbf{r}, 0^-) = \mathcal{E}_i(t, \mathbf{r}, 0) = E_i(t, \mathbf{r})}, \tag{4c}$$

and,

$$\boxed{\mathcal{B}_{z,+}(t, \mathbf{r}, 0^+) = \mathcal{B}_{z,-}(t, \mathbf{r}, 0^-) = \mathcal{B}_z(t, \mathbf{r}, 0) = B_z(t, \mathbf{r})}, \tag{4d}$$

which are continuous across the interface and denote the fields that interact with the charge at $z = 0$.

2D linear response theory

Generically, the 2D current density can be expressed in terms of polarization and magnetization densities,

$$J_i(t,\mathbf{r}) = \partial_t P_i(t,\mathbf{r}) + c\epsilon_{ij}\partial^j M_z(t,\mathbf{r}), \qquad \rho(t,\mathbf{r}) = -\partial_i P^i(t,\mathbf{r}). \tag{5}$$

For simplicity, we assume the material is non-magnetic $M_z = 0$, such that polarization density $\mathbf{P}(t,\mathbf{r})$ only couples to the in-plane electric field $\mathbf{E}(t,\mathbf{r}) = \vec{\mathcal{E}}_\parallel(t,\mathbf{r},0)$ at $z=0$. In real space, the linear response is,

$$P_i(t,\mathbf{r}) = \int_{-\infty}^{t} dt' \int d\mathbf{r}' \chi_{ij}(t-t',\mathbf{r},\mathbf{r}') E^j(t',\mathbf{r}'). \tag{6}$$

Due to translational symmetry in time, we can always convert to the frequency ω space,

$$P_i(\omega,\mathbf{r}) = \int d\mathbf{r}' \chi_{ij}(\omega,\mathbf{r},\mathbf{r}') E^j(\omega,\mathbf{r}'). \tag{7}$$

However, the response function χ depends on the boundary conditions in $\mathbf{r}$ and is therefore fundamentally different for the bulk and edge. Here, we only consider an infinite (bulk) medium such that $\chi_{ij}(\omega,\mathbf{r},\mathbf{r}') = \chi_{ij}(\omega,\mathbf{r}-\mathbf{r}')$. In this case, the in-plane momentum $\mathbf{q} = (q_x, q_y)$ is conserved and we can Fourier transform to the reciprocal space as,

$$P_i(\omega,\mathbf{q}) = \chi_{ij}(\omega,\mathbf{q}) E^j(\omega,\mathbf{q}), \qquad \chi_{ij}(\omega,\mathbf{r}-\mathbf{r}') = \int \frac{d\mathbf{q}}{(2\pi)^2} \chi_{ij}(\omega,\mathbf{q}) e^{i\mathbf{q}\cdot(\mathbf{r}-\mathbf{r}')}, \tag{8}$$

where we have implicitly assumed the medium is continuous (translational symmetry). Hence, the boundary condition on the electric displacement field amounts to,

$$\boxed{\Delta\mathcal{D}_z(\omega,\mathbf{q}) = -i4\pi q^i \chi_{ij}(\omega,\mathbf{q}) E^j(\omega,\mathbf{q})}, \tag{9a}$$

while the boundary conditions on the auxiliary magnetic fields are,

$$\boxed{\epsilon_{ij}\Delta\mathcal{H}^j(\omega,\mathbf{q}) = i4\pi\frac{\omega}{c}\chi_{ij}(\omega,\mathbf{q}) E^j(\omega,\mathbf{q})}. \tag{9b}$$

This gives the complete set of equations needed to specify the electromagnetic field in the bulk medium.

Supplementary Note 2: Navier-Stokes equations of the viscous Hall fluid

We consider a 2D electron fluid [2–5], such as graphene, subject to a time-varying electromagnetic field. The dynamics of a classical fluid are governed by the famous Navier-Stokes equations which fundamentally describe *viscous* flow. These equations determine the spatiotemporal evolution of the velocity field $\mathbf{v} = (v_x, v_y)$ and the number density n to external forces and internal pressures,

$$mn\left[\partial_t \mathbf{v} + (\mathbf{v}\cdot\boldsymbol{\nabla})\mathbf{v} + \gamma\mathbf{v}\right] = -\boldsymbol{\nabla}p + \mathbf{f} + \boldsymbol{\nabla}\cdot\varsigma. \tag{10}$$

Here, m is the effective mass of the electron [6] and $\boldsymbol{\nabla} = (\partial_x, \partial_y)$ is the 2D del operator. Combining Supplementary Eq. (10) with the continuity relation provides the complete set of equations needed to specify $\mathbf{v}$ and n with appropriate boundary conditions,

$$\frac{\partial n}{\partial t} + \boldsymbol{\nabla}\cdot(n\mathbf{v}) = 0. \tag{11}$$

$\mathbf{r} = (x, y)$ labels in-plane coordinates. Additionally, these quantities are directly proportional to the 2D charge ρ and current $\mathbf{J} = (J_x, J_y)$ densities via,

$$\rho = -en, \qquad \mathbf{J} = -en\mathbf{v} = \rho\mathbf{v}, \tag{12}$$

where e is the elementary charge of the electron. Notice that charge is conserved $\partial_t \rho + \boldsymbol{\nabla} \cdot \mathbf{J} = 0$ due to the continuity equation [Supplementary Eq. (11)].

We will walk through each term in Supplementary Eq. (10) sequentially to understand its physical significance. $\partial_t \mathbf{v}$ is the acceleration denoting the rate of change of the velocity field. The nonlinear term $(\mathbf{v} \cdot \boldsymbol{\nabla})\mathbf{v}$ is known as the convective acceleration arising from nonuniform fluid flow, but can be neglected at linear order. The damping term $\gamma\mathbf{v}$ is proportional to the phenomenological transport time $\gamma = \tau^{-1}$, characterising momentum-non-conserving collisions. p is the thermodynamic pressure, related to fluctuations in the internal energy of the fluid, and $\mathbf{f} = (f_x, f_y)$ is the external Lorentz force,

$$\mathbf{f} = \rho\mathbf{E} + \frac{1}{c}\mathbf{J} \times (B\hat{z}) = -en\left(\mathbf{E} + B\frac{\mathbf{v}}{c} \times \hat{z}\right), \tag{13}$$

where c is the speed of light. Note that $\mathbf{E}(t, \mathbf{r}) = \vec{\mathcal{E}}_{||}(t, \mathbf{r}, 0)$ represents the parallel electric field strictly at the location of the 2D electron fluid $z = 0$. B is a uniform magnetic field that induces cyclotron motion. Finally, we arrive at the last contribution $\bar{\varsigma}$, the rank-two viscous stress tensor. The divergence of the stress $\boldsymbol{\nabla} \cdot \bar{\varsigma}$ describes the restoring force generated by deformations in the fluid.

In two dimensions, the stress tensor can be expressed in terms of the rank-four viscosity tensor $\eta_{ij,kl}$ and the velocity gradient (strain-rate) tensor $(\partial^i v^j + \partial^j v^i)/2$,

$$\varsigma_{ij} = \frac{1}{2}\eta_{ij,kl}(\partial^k v^l + \partial^l v^k). \tag{14}$$

Repeated spatial indices $(i = x, y)$ implies summation. Viscosity is a measure of a fluid's resistance to deformation. In a 2D isotropic fluid, there are three possible contributions to the viscosity tensor,

$$\eta_{ij,kl} = \zeta\delta_{ij}\delta_{kl} + \eta(\delta_{ik}\delta_{jl} + \delta_{il}\delta_{jk} - \delta_{ij}\delta_{kl}) - \eta_H(\delta_{jk}\epsilon_{il} - \delta_{il}\epsilon_{kj}). \tag{15}$$

ζ and η are the bulk and shear viscosities [7–9] respectively, which govern dissipation due to compression and shearing. In 2D, the viscous stress tensor can then be expressed in compact Pauli notation [10],

$$\varsigma = \zeta\boldsymbol{\nabla} \cdot \mathbf{v} + (\eta - i\eta_H\tau_y)\left[(\partial_x v_x - \partial_y v_y)\tau_z + (\partial_x v_y + \partial_y v_x)\tau_x\right], \tag{16}$$

where the Pauli matrices τ_i act on Cartesian coordinates. From the second law of thermodynamics, $\zeta \geq 0$ and $\eta \geq 0$ must always be positive to ensure lossy fluid flow. Generally, ζ and η can also be frequency dependent but in undoped graphene [11], the bulk viscosity vanishes $\zeta = 0$ and the shear viscosity η is a very small constant. One additional viscous component is permitted in parity and time-reversal breaking fluids – the so-called Hall viscosity η_H [12–15]. Hall viscosity, also known as odd viscosity in fluid mechanics [16–19], can be either positive or negative as it is dissipationless. The stress response due to η_H generates a force perpendicular to an applied strain and therefore does no work. In most cases – in fact the topologically nontrivial cases – Hall viscosity acts as a repulsion to the applied magnetic field B which leads to very intriguing phenomena.

Magnetohydrodynamic linear response theory

Within the regimes we are interested, the amplitude of the perturbing electric field $\mathbf{E}$ is relatively weak. Therefore, the fluctuations in the number density δn can be linearized around its equilibrium value $n(t, \mathbf{r}) = n_0 + \delta n(t, \mathbf{r})$. The equilibrium

number density n_0 (electron concentration) is assumed large compared to the fluctuations $|\delta n|/n_0 \ll 1$. This also implies the velocity field $|\mathbf{v}|/v_F \ll 1$ is comparably small relative to the Fermi velocity v_F. Hence, we may neglect the nonlinear convective acceleration and linearize the pressure component $\nabla p = (\mathfrak{B}/n_0)\nabla n$, where $\mathfrak{B}$ is the bulk modulus [20] and determines the speed of sound in the 2D electron fluid $v_s^2 = \mathfrak{B}/(mn_0) = v_F^2/2$. We have assumed linear dispersion characteristic of graphene [21, 22] to obtain the proportionality of v_s to the Fermi velocity v_F. Inserting the charge density $\rho = -en$ and linearized current density $\mathbf{J} = -en_0\mathbf{v}$ into Supplementary Eq. (10), we obtain the linear form of the viscous hydrodynamic model,

$$\boxed{\partial_t \mathbf{J} = -v_s^2 \nabla\rho - (\gamma - \nu\nabla^2)\mathbf{J} - (\omega_c + \nu_H\nabla^2)\mathbf{J}\times\hat{z} + \frac{e^2 n_0}{m}\mathbf{E}}, \tag{17}$$

along with the continuity equation $\partial_t\rho + \nabla\cdot\mathbf{J} = 0$. Here, $D_\nu = \sqrt{\nu\tau} = \sqrt{\nu/\gamma}$ and $D_H = \sqrt{\nu_H/\omega_c}$ are the diffusion lengths. Note, the charge couples strictly to the in-plane electric field $\mathbf{E}(t,\mathbf{r}) = \vec{\mathcal{E}}_{||}(t,\mathbf{r},0)$ at the location of the surface charge $z = 0$. In a semiclassical electron fluid, the kinetic viscosities ν and ν_H are related [10, 14],

$$\nu = \frac{B_0^2}{B^2 + B_0^2}\nu_0, \qquad \nu_H = \frac{BB_0}{B^2 + B_0^2}\nu_0, \tag{18}$$

where $\nu_0 \geq 0$ is the kinematic shear viscosity at zero bias $B = 0$, and $B_0 = cn_0/(4\pi e\mathcal{N}_0\nu_0)$ is an intrinsic magnetic field of the electron fluid. $\mathcal{N}_0$ being the density of states at the Fermi energy. Notice that the product of ω_c and ν_H is positive definite $\omega_c\nu_H > 0$; the Hall viscosity repels the magnetic field for all values of B. The Hall diffusion length D_H is therefore a real mesoscopic scale of the charged viscous Hall fluid. In the dissipationless limit $\gamma \to$ and $\nu \to 0$, we obtain the hydrodynamic model of an ideal quantum Hall fluid.

To derive the linear response theory, we transfer to the reciprocal space and use the continuity equation $\omega\rho = \mathbf{q}\cdot\mathbf{J}$. In terms of ω and $\mathbf{q}$ we have,

$$-i\omega J_i = -i\frac{v_s^2}{\omega}q_i q^j J_j - \Gamma J_i - \Omega_c\epsilon_{ij}J^j + \frac{e^2 n_0}{m}E_i, \tag{19a}$$

where $\Gamma(q) = \gamma + \nu q^2$ is the viscous damping rate and $\Omega_c(q) = \omega_c - \nu_H q^2$ is the viscous cyclotron frequency. Rearranging we obtain,

$$[\omega(-i\omega + \Gamma)\delta_{ij} + \omega\Omega_c\epsilon_{ij} + iv_s^2 q_i q_j]J^j = \omega\frac{e^2 n_0}{m}E^i, \tag{19b}$$

which gives,

$$J_i = \frac{e^2 n_0}{m}\frac{[\omega[(-i\omega + \Gamma)\delta_{ij} - \Omega_c\epsilon_{ij}] + iv_s^2(q^2\delta_{ij} - q_i q_j)]E^j}{\omega[(-i\omega + \Gamma)^2 + \Omega_c^2] + v_s^2 q^2(\omega + i\Gamma)}. \tag{19c}$$

Using $P_i = iJ_i/\omega$,

$$P_i = \frac{e^2 n_0}{m}\frac{[-(\omega + i\Gamma)\delta_{ij} + i\Omega_c\epsilon_{ij} + \omega^{-1}v_s^2(q^2\delta_{ij} - q_i q_j)]E^j}{\omega[(\omega + i\Gamma)^2 - \Omega_c^2] - v_s^2 q^2(\omega + i\Gamma)} = \chi_{ij}E^j. \tag{19d}$$

The susceptibility tensor in reciprocal space is then,

$$\boxed{\chi_{ij}(\omega,\mathbf{q}) = \chi^T(\omega,q)(\delta_{ij} - \hat{q}_i\hat{q}_j) + \chi^L(\omega,q)\hat{q}_i\hat{q}_j + ig(\omega,q)\epsilon_{ij},} \tag{20a}$$

where,

$$\boxed{\chi^T = -\frac{e^2 n_0/m}{\omega\tilde{\omega}}\left(1 + \frac{\omega\Omega_c^2}{\omega(\tilde{\omega}^2 - \Omega_c^2) - v_s^2 q^2\tilde{\omega}}\right), \qquad \chi^L = -\frac{(e^2 n_0/m)\tilde{\omega}}{\omega(\tilde{\omega}^2 - \Omega_c^2) - v_s^2 q^2\tilde{\omega}}, \qquad g = \frac{(e^2 n_0/m)\Omega_c}{\omega(\tilde{\omega}^2 - \Omega_c^2) - v_s^2 q^2\tilde{\omega}}.} \tag{20b}$$

Here, $\tilde{\omega} = \omega + i\Gamma$ is the dissipative frequency. In the lossless limit $\Gamma \to 0$ we obtain the linear response theory of an ideal quantum Hall fluid,

$$\chi^T = -\frac{e^2 n_0/m}{\omega^2}\left(1 + \frac{\Omega_c^2}{\omega^2 - \Omega_c^2 - v_s^2 q^2}\right), \qquad \chi^L = -\frac{e^2 n_0/m}{\omega^2 - \Omega_c^2 - v_s^2 q^2}, \qquad g = \frac{(e^2 n_0/m)\Omega_c}{\omega(\omega^2 - \Omega_c^2 - v_s^2 q^2)}. \tag{20c}$$

We will now prove that the viscous Hall fluid is topologically nontrivial in the quantum limit.

Supplementary Note 3: Optical N-invariant

We utilize the Green's function (response function) formalism popularized by Volovik [23] and Gurarie [24] to evaluate the optical N-invariant. This approach is very powerful because it is naturally generalized to dissipative and finite temperature systems [25]. Since χ is precisely the Green's function of the polarization density $\mathbf{P}$, it possesses a topological index,

$$\boxed{N = \frac{1}{24\pi^2}\int d^3q\, \mathcal{F}(\Omega, \mathbf{q}), \qquad \mathcal{F} = \epsilon^{\alpha\beta\gamma}\mathrm{tr}\left[\chi\frac{\partial\chi^{-1}}{\partial q_\alpha}\chi\frac{\partial\chi^{-1}}{\partial q_\beta}\chi\frac{\partial\chi^{-1}}{\partial q_\gamma}\right]}. \tag{21}$$

$q_\alpha = (\Omega, \mathbf{q})$ is the 2+1D momentum and $d^3q = d\Omega d\mathbf{q}$ is the total volume element in reciprocal space. Note that we let $\omega \to \Omega$ be a complex parameter and analytically continue χ into the complex plane. The integral of Ω is performed vertically over all imaginary (Matsubara) frequencies $\Im(\Omega) \in (-i\infty, +i\infty)$ and within a spectral gap $0 < \hbar\omega < E_{\mathrm{bg}}$, where $\omega = \Re(\Omega)$. It is not obvious that this contour is convergent and nondegenerate so we will provide a short proof.

First we need to prove the infinite line integral over Ω is regularized and topologically equivalent to the circle S^1 in the dissipationless limit. Consider the closed left-handed hemispherical contour of radius R in the complex plane. Applying the residue theorem gives,

$$-2\pi i\sum_{\Omega_i\in\mathbf{\Omega}}\mathrm{res}[\mathcal{F}(\Omega_i)] = \oint d\Omega\mathcal{F}(\Omega) = \int_{\omega-iR}^{\omega+iR} d\Omega\mathcal{F}(\Omega) + \int_{\mathrm{arc}} d\Omega\mathcal{F}(\Omega), \tag{22}$$

where ω is the photon energy in the gap $0 < \hbar\omega < E_{\mathrm{bg}}$ and $\int_{\mathrm{arc}}$ is the integral over the arc. The arc radius R is large enough to encircle all poles $\Omega_i \in \mathbf{\Omega}$ in the right complex plane, where $\mathbf{\Omega}$ is the set of poles bounded by the contour. Taking $|\Omega| \to \infty$ we know from the f-sum rule that,

$$\lim_{|\Omega|\to\infty}\chi_{ij}(\Omega, \mathbf{q}) \to -\frac{e^2 n_0}{m\Omega^2}\delta_{ij}, \tag{23}$$

which is independent of $\mathbf{q}$ and $\det\chi \neq 0$. We also assume the response function is regularized at $q \to \infty$,

$$\lim_{q\to\infty}\chi(\Omega, \mathbf{q}) \to \chi(\Omega, q), \tag{24}$$

which will be exploited in the following sections. Since the identity $\delta_{ij} > 0$ is positive definite for all values of $\mathbf{q}$, the path is nondegenerate for large enough contours in the complex plane [26]. In the asymptotic limit $R \to \infty$, the temporal component approaches the identity multiplied by a simple pole $\lim_{|\Omega|\to\infty}\chi\partial_\Omega\chi^{-1} \propto \mathbb{1}_2/\Omega$, since $\det\chi \neq 0$ is invertible. The arc integral is then proportional to,

$$\lim_{|\Omega|\to\infty}\int_{\mathrm{arc}} d\Omega\mathcal{F}(\Omega) \propto \lim_{|\Omega|\to\infty}\int_{\mathrm{arc}}\frac{d\Omega}{\Omega}\epsilon^{ij}\mathrm{tr}\left[\chi\partial_i\chi^{-1}\chi\partial_j\chi^{-1}\right] \to 0. \tag{25}$$

The arc integral vanishes because χ is independent of $\mathbf{q}$ in the asymptotic limit and therefore $\lim_{|\Omega|\to\infty}\chi\partial_i\chi^{-1} \to 0$ since again, $\det\chi \neq 0$ is invertible. Hence, the imaginary line integral is convergent,

$$-2\pi i\sum_{\Omega_i\in\mathbf{\Omega}}\mathrm{res}[\mathcal{F}(\Omega_i)] = \int_{\omega-i\infty}^{\omega+i\infty} d\Omega\mathcal{F}(\Omega), \tag{26}$$

which defines the homotopy of a circle S^1 when including the point at $|\Omega| = \infty$.

Homotopy equivalence of susceptibility and conductivity

Here we prove that even with dissipation, N is identical under the substitution $\chi = i\sigma/\Omega$,

$$\mathcal{F} = \epsilon^{\alpha\beta\gamma}\mathrm{tr}\left[\sigma\frac{\partial\sigma^{-1}}{\partial q_\alpha}\sigma\frac{\partial\sigma^{-1}}{\partial q_\beta}\sigma\frac{\partial\sigma^{-1}}{\partial q_\gamma}\right] + \delta\mathcal{F}, \qquad \delta\mathcal{F} = 3\Omega^{-1}\epsilon^{ij}\mathrm{tr}\left[\sigma\frac{\partial\sigma^{-1}}{\partial q_i}\sigma\frac{\partial\sigma^{-1}}{\partial q_j}\right], \tag{27a}$$

where we have used the cyclic properties of the trace. Now we use $\sigma\partial_i\sigma^{-1}\sigma = -\partial_i\sigma$ to rewrite $\delta\mathcal{F}$ as,

$$\delta\mathcal{F} = -3\Omega^{-1}\epsilon^{ij}\mathrm{tr}\left[\partial_i\sigma\partial_j\sigma^{-1}\right] = -3\Omega^{-1}\epsilon^{ij}\partial_i\mathrm{tr}\left[\sigma\partial_j\sigma^{-1}\right], \tag{27b}$$

where we have used the properties of the antisymmetric tensor. We are left with a total divergence in the wave vector $\mathbf{q}$, which in polar coordinates is,

$$\delta N = \frac{-1}{8\pi^2}\int\frac{d\Omega}{\Omega}\int_S d\mathbf{q}\,\epsilon^{ij}\partial_i\mathrm{tr}\left[\sigma\partial_j\sigma^{-1}\right] = \frac{-1}{8\pi^2}\int\frac{d\Omega}{\Omega}\oint_{\partial S} dq_i\epsilon^{ij}\mathrm{tr}\left[\sigma\partial_j\sigma^{-1}\right] = \frac{-1}{8\pi^2}\int\frac{d\Omega}{\Omega}\lim_{q\to\infty}\oint d\phi\mathrm{tr}\left[\sigma\partial_\phi\sigma^{-1}\right] = 0. \tag{28}$$

The response function is continuous in $\mathbf{q}$, even in the presence of dissipation. Assuming $\lim_{q\to\infty}\sigma(\Omega,\mathbf{q}) \to \sigma(\Omega, q)$ is regularized in the continuum theory, the surface integral vanishes from the divergence theorem since $\lim_{q\to\infty}\sigma\partial_\phi\sigma^{-1} = 0$ is independent of ϕ. Hence, N can be equivalently defined through the susceptibility χ or conductivity σ tensor. The same conclusion holds in the lattice case due to periodic boundary conditions.

Topological quantization and effect of dissipation

Now we show the effects of dissipation on N and determine when the invariant is topologically quantized (immune to perturbations). Consider a variation in the response function $\chi \to \chi + \delta\chi$, which results in,

$$\chi\partial_\alpha\chi^{-1} \to \chi\partial_\alpha\chi^{-1} + \delta\chi\partial_\alpha\chi^{-1} + \chi\partial_\alpha(\delta\chi)^{-1} = \chi\partial_\alpha\chi^{-1} - \chi\partial_\alpha\chi^{-1}\delta\chi\chi^{-1} - \partial_\alpha\delta\chi\chi^{-1} \tag{29a}$$

where we have utilized $\delta\chi^{-1} = -\chi^{-1}\delta\chi\chi^{-1}$. To first-order in $\delta\chi$ we have,

$$\begin{aligned}\delta\mathcal{F} &= -3\epsilon^{\alpha\beta\gamma}\mathrm{tr}\left[\chi\partial_\alpha\chi^{-1}\delta\chi\partial_\beta\chi^{-1}\chi\partial_\gamma\chi^{-1}\right] - 3\epsilon^{\alpha\beta\gamma}\mathrm{tr}\left[\partial_\alpha\delta\chi\partial_\beta\chi^{-1}\chi\partial_\gamma\chi^{-1}\right]\\ &= -3\epsilon^{\alpha\beta\gamma}\mathrm{tr}\left[\partial_\alpha\chi^{-1}\delta\chi\partial_\beta\chi^{-1}\chi\partial_\gamma\chi^{-1}\chi\right] - 3\epsilon^{\alpha\beta\gamma}\mathrm{tr}\left[\chi^{-1}\partial_\alpha\delta\chi\partial_\beta\chi^{-1}\chi\partial_\gamma\chi^{-1}\chi\right]\end{aligned} \tag{29b}$$

This can be expressed as one derivative by the product rule,

$$\delta\mathcal{F} = -3\epsilon^{\alpha\beta\gamma}\mathrm{tr}\left[\partial_\alpha(\chi^{-1}\delta\chi)\partial_\beta\chi^{-1}\chi\partial_\gamma\chi^{-1}\chi\right], \tag{29c}$$

and then changing the order of the derivative,

$$\delta\mathcal{F} = -3\epsilon^{\alpha\beta\gamma}\partial_\alpha\mathrm{tr}\left[\chi^{-1}\delta\chi\partial_\beta\chi^{-1}\chi\partial_\gamma\chi^{-1}\chi\right] + 3\epsilon^{\alpha\beta\gamma}\mathrm{tr}\left[\chi^{-1}\delta\chi\partial_\alpha(\partial_\beta\chi^{-1}\chi\partial_\gamma\chi^{-1}\chi)\right]. \tag{29d}$$

Using the antisymmetric properties of the Levi-Civita symbol, the second term can be shown to reduce to zero,

$$\begin{aligned}&\epsilon^{\alpha\beta\gamma}\mathrm{tr}\left[\chi^{-1}\delta\chi\partial_\alpha(\partial_\beta\chi^{-1}\chi)\partial_\gamma\chi^{-1}\chi\right] + \epsilon^{\alpha\beta\gamma}\mathrm{tr}\left[\chi^{-1}\delta\chi\partial_\beta\chi^{-1}\chi\partial_\alpha(\partial_\gamma\chi^{-1}\chi)\right]\\ &= \epsilon^{\alpha\beta\gamma}\mathrm{tr}\left[\delta\chi\partial_\beta\chi^{-1}\partial_\alpha\chi\partial_\gamma\chi^{-1}\right] + \epsilon^{\alpha\beta\gamma}\mathrm{tr}\left[\chi^{-1}\delta\chi\partial_\beta\chi^{-1}\chi\partial_\gamma\chi^{-1}\partial_\alpha\chi\right]\\ &= \epsilon^{\alpha\beta\gamma}\mathrm{tr}\left[\delta\chi\partial_\beta\chi^{-1}\partial_\alpha\chi\partial_\gamma\chi^{-1}\right] + \epsilon^{\alpha\beta\gamma}\mathrm{tr}\left[\delta\chi\partial_\beta\chi^{-1}\chi\partial_\gamma\chi\partial_\alpha\chi^{-1}\right] = 0.\end{aligned} \tag{29e}$$

Hence, the variation in δN due to $\delta\mathcal{F}$ is,

$$\boxed{\delta N = -\frac{\epsilon^{\alpha\beta\gamma}}{8\pi^2}\int d^3q\,\partial_\alpha \mathrm{tr}\left[\delta\chi\partial_\beta\chi^{-1}\chi\partial_\gamma\chi^{-1}\right]}. \tag{30}$$

In the absence of dissipation $\Gamma \to 0$, the susceptibility is a continuous function along the $\Im(\Omega)$ axis. By the divergence theorem, $\delta N = 0$ vanishes as long as the integrand vanishes sufficiently fast on the boundary at infinity. Due to the f-sum rule, the temporal boundaries vanish which was proven in the previous sections. The remaining boundary term in polar coordinates reads,

$$\begin{aligned}\delta N &= \frac{1}{8\pi^2}\int d\Omega d\phi dq\partial_q \mathrm{tr}[\delta\chi(\partial_\Omega\chi^{-1}\chi\partial_\phi\chi^{-1} - \partial_\phi\chi^{-1}\chi\partial_\Omega\chi^{-1})] \\ &= \frac{1}{8\pi^2}\int d\Omega d\phi \lim_{q\to\infty}\mathrm{tr}[\delta\chi(\partial_\Omega\chi^{-1}\chi\partial_\phi\chi^{-1} - \partial_\phi\chi^{-1}\chi\partial_\Omega\chi^{-1})] \to 0\end{aligned} \tag{31}$$

Since the response function $\lim_{q\to\infty}\chi(\Omega,\mathbf{q}) \to \chi(\Omega,q)$ is regularized in the continuum theory, the surface integral vanishes because $\lim_{q\to\infty}\chi\partial_\phi\chi^{-1} \to 0$ is independent of ϕ. As a consequence, the 2D momentum space is topologically equivalent to the sphere $\mathbb{R}^2 \simeq S^2$ and the N-invariant is quantized $N \in \mathbb{Z}$.

However, when $\Gamma \neq 0$, the requirement of causality introduces a discontinuity along the $\Im(\Omega)$ axis due to $\Gamma \to \mathrm{sgn}[\Im(\Omega)]\Gamma$ [25]. This means contributions from temporal boundaries are permitted,

$$\delta N = -\frac{\epsilon^{ij}}{8\pi^2}\int d^3q\,\partial_\Omega \mathrm{tr}\left[\delta\chi\partial_i\chi^{-1}\chi\partial_j\chi^{-1}\right] \neq 0, \tag{32}$$

but the boundary terms over $\mathbf{q}$ vanish because the response function is regularized $\lim_{q\to\infty}\chi(\Omega,\mathbf{q}) \to \chi(\Omega,q)$. Hence, N is not quantized in lossy systems but can still be evaluated through the aforementioned recipe.

Optical N-invariant of the viscous Hall fluid

First note, using properties of the trace and the antisymmetric tensor, we can express $\mathcal{F}$ as,

$$\begin{aligned}\mathcal{F} &= 3\epsilon^{ij}\mathrm{tr}\left[\chi\partial_\Omega\chi^{-1}\chi\partial_i\chi^{-1}\chi\partial_j\chi^{-1}\right] \\ &= -3\epsilon^{ij}\mathrm{tr}\left[\chi\partial_\Omega\chi^{-1}\partial_i\chi\partial_j\chi^{-1}\right] = -3q^{-1}\mathrm{tr}\left[\chi\partial_\Omega\chi^{-1}(\partial_q\chi\partial_\phi\chi^{-1} - \partial_\phi\chi\partial_q\chi^{-1})\right]\end{aligned} \tag{33a}$$

and we have transformed to polar coordinates. In this case we have,

$$\chi_{ij} = -\frac{e^2n_0/m}{\Omega\Delta}\left[(\Omega\tilde{\Omega} - v_s^2q^2)\hat{\varphi}_i\hat{\varphi}_j + \Omega\tilde{\Omega}\hat{q}_i\hat{q}_j - i\Omega\Omega_c\epsilon_{ij}\right], \qquad \Delta = \tilde{\Omega}A - \Omega\Omega_c^2, \qquad A = \Omega\tilde{\Omega} - v_s^2q^2 \tag{33b}$$

which has the derivatives,

$$\frac{1}{q}\partial_\phi\chi_{ij} = -\frac{e^2n_0/m}{\Omega\Delta}v_s^2q(\hat{\varphi}_i\hat{q}_j + \hat{q}_i\hat{\varphi}_j), \tag{33c}$$

$$\begin{aligned}\partial_q\chi_{ij} =& \frac{e^2n_0/m\Delta'}{\Omega\Delta^2}\left[(\Omega\tilde{\Omega} - v_s^2q^2)\hat{\varphi}_i\hat{\varphi}_j + \Omega\tilde{\Omega}\hat{q}_i\hat{q}_j - i\Omega\Omega_c\epsilon_{ij}\right] - \frac{e^2n_0/m}{\Omega\Delta}\left[(\Omega\tilde{\Omega} - v_s^2q^2)'\hat{\varphi}_i\hat{\varphi}_j + \Omega\tilde{\Omega}'\hat{q}_i\hat{q}_j - i\Omega\Omega_c'\epsilon_{ij}\right] \\ =& \frac{e^2n_0/m}{\Omega\Delta^2}\left[(\Delta'A - \Delta A)\hat{\varphi}_i\hat{\varphi}_j + \Omega(\Delta'\tilde{\Omega} - \tilde{\Omega}'\Delta)\hat{q}_i\hat{q}_j - i\Omega(\Delta'\Omega_c - \Delta\Omega_c')\epsilon_{ij}\right] \\ =& \frac{e^2n_0/m}{\Omega\Delta^2}\Big\{[\tilde{\Omega}'A^2 + \Omega(\Omega_c^2A' - \Omega_c^{2\prime}A)]\hat{\varphi}_i\hat{\varphi}_j + [\Omega\tilde{\Omega}A' + \Omega^2(\Omega_c^2\tilde{\Omega}' - \Omega_c^{2\prime}\tilde{\Omega})]\hat{q}_i\hat{q}_j \\ & - i\Omega[\Omega_c(\tilde{\Omega}A)' - \Omega_c'(\tilde{\Omega}A) - \Omega\Omega_c'\Omega_c^2]\epsilon_{ij}\Big\}\end{aligned} \tag{33d}$$

This inverse susceptibility tensor is found via,

$$\chi^{-1}_{ij} = -\frac{m}{e^2 n_0}\left[\Omega\tilde{\Omega}\hat{\varphi}_i\hat{\varphi}_j + (\Omega\tilde{\Omega} - v_s^2 q^2)\hat{q}_i\hat{q}_j + i\Omega\Omega_c\epsilon_{ij}\right] \tag{33e}$$

Which gives the derivatives,

$$\partial_\Omega \chi^{-1}_{ij} = -\frac{m}{e^2 n_0}[(\Omega + \tilde{\Omega})\delta_{ij} + i\Omega_c\epsilon_{ij}], \qquad \frac{1}{q}\partial_\phi \chi^{-1}_{ij} = \frac{m v_s^2 q}{e^2 n_0}(\hat{\varphi}_i\hat{q}_j + \hat{q}_i\hat{\varphi}_j), \tag{33f}$$

$$\partial_q \chi^{-1}_{ij} = -\frac{m}{e^2 n_0}\left[\Omega\tilde{\Omega}'\hat{\varphi}_i\hat{\varphi}_j + (\Omega\tilde{\Omega} - v_s^2 q^2)'\hat{q}_i\hat{q}_j + i\Omega\Omega_c'\epsilon_{ij}\right] \tag{33g}$$

The temporal derivative is,

$$\chi_{ik}\partial_\Omega\chi^{-1}_{kj} = \frac{1}{\Omega\Delta}\left\{[\Delta + \Omega(\tilde{\Omega}\Omega - v_s^2 q^2)]\delta_{ij} + (\Omega + \tilde{\Omega})v_s^2 q_i q_j + i v_s^2 \Omega_c q^2 \hat{q}_i\hat{\varphi}_j - i(\Omega^2 + v_s^2 q^2)\Omega_c\epsilon_{ij}\right\} \tag{33h}$$

while the spatial derivatives are,

$$\begin{aligned}
\frac{1}{q}(\partial_q\chi_{ik}\partial_\phi\chi^{-1}_{kj} - \partial_\phi\chi_{ik}\partial_q\chi^{-1}_{kj}) &= \frac{v_s^2 q}{\Omega\Delta^2}\left[(\Delta' A - A'\Delta)\hat{\phi}_i\hat{q}_j + \Omega(\Delta'\tilde{\Omega} - \tilde{\Omega}'\Delta)\hat{q}_i\hat{\phi}_j + i\Omega(\Delta'\Omega_c - \Delta\Omega_c')(\hat{\phi}_i\hat{\phi}_j - \hat{q}_i\hat{q}_j)\right] \\
&- \frac{v_s^2 q}{\Omega\Delta}[A'\hat{\phi}_i\hat{q}_j + \Omega\tilde{\Omega}'\hat{q}_i\hat{\phi}_j + i\Omega\Omega_c'(\hat{\phi}_i\hat{\phi}_j - \hat{q}_i\hat{q}_j)] \\
&= \frac{v_s^2 q}{\Omega\Delta^2}\left[(\Delta' A - 2A'\Delta)\hat{\phi}_i\hat{q}_j + \Omega(\Delta'\tilde{\Omega} - 2\tilde{\Omega}'\Delta)\hat{q}_i\hat{\phi}_j + i\Omega(\Delta'\Omega_c - 2\Delta\Omega_c')(\hat{\phi}_i\hat{\phi}_j - \hat{q}_i\hat{q}_j)\right]
\end{aligned} \tag{33i}$$

The trace is then,

$$\begin{aligned}
\mathcal{F} &= -\frac{3v_s^2 q}{\Omega^2\Delta^3}[-i\Omega(\Omega + \tilde{\Omega})v_s^2 q^2(\Delta'\Omega_c - 2\Delta\Omega_c') + i v_s^2 q^2\Omega_c(\Delta' A - 2A'\Delta) \\
&- i\Omega_c(\Omega^2 + v_s^2 q^2)(\Delta' A - 2A'\Delta - \Omega\Delta'\tilde{\Omega} + 2\Omega\tilde{\Omega}'\Delta)] \\
&= -i\frac{3v_s^2 q}{\Omega^2\Delta^3}[v_s^2 q^2\Omega_c\Delta'(A - \Omega(\Omega + \tilde{\Omega})) + 2v_s^2 q^2\Delta(\Omega_c'\Omega(\Omega + \tilde{\Omega}) - A'\Omega_c) \\
&+ \Omega_c(\Omega^2 + v_s^2 q^2)(\Delta' v_s^2 q^2 - 2\Delta(v_s^2 q^2)')] \\
&= -i\frac{6v_s^2 q}{\Omega^2\Delta^3}[v_s^2 q^2\Delta\Omega_c'\Omega(\Omega + \tilde{\Omega}) - \Omega_c\Delta\Omega(\tilde{\Omega}' v_s^2 q^2 + \Omega(v_s^2 q^2)')] \\
&= -i\frac{6v_s^2 q^2}{\Omega\Delta^2}[q(\Omega + \tilde{\Omega})\Omega_c' - (2\Omega + q\tilde{\Omega}')\Omega_c]
\end{aligned} \tag{33j}$$

which gives,

$$\mathcal{F} = -i\frac{6v_s^2 q^2}{\Omega}\frac{[q(\Omega + \tilde{\Omega})\Omega_c' - (2\Omega + q\tilde{\Omega}')\Omega_c]}{[\Omega(\tilde{\Omega}^2 - \Omega_c^2) - v_s^2 q^2\tilde{\Omega}]^2} \tag{34}$$

Inserting $\tilde{\Omega} = \Omega + i\Gamma$ with $\Gamma = \gamma + \nu q^2$ and $\Omega_c = \omega_c - \nu_H q^2$, we arrive at,

$$\boxed{\mathcal{F} = -12i\frac{v_s^4 q^2}{\Omega}\frac{[\Omega(\omega_c + \nu_H q^2) + i(\gamma\nu_H + \omega_c\nu)q^2]}{[\Omega(\tilde{\Omega}^2 - \Omega_c^2) - v_s^2 q^2\tilde{\Omega}]^2}}, \tag{35}$$

with the implication that $\Gamma \to \text{sgn}[\Im(\Omega)]\Gamma$ has the same sign as the Matsubara frequency. With dissipation, the integral can be evaluated numerically for arbitrary imaginary contours, but N is not quantized. It is quantized, however, for quantum systems $\Gamma = 0$. In the dissipationless limit we have,

$$\mathcal{F} = -12i\frac{v_s^4 q^2}{\Omega^2}\frac{\omega_c + \nu_H q^2}{(\Omega^2 - \Omega_c^2 - v_s^2 q^2)^2}, \tag{36}$$

Note that $\mathcal{F}$ is singular at $\Omega = 0$ and whenever $\Re(\Omega) \geq \min[\sqrt{\Omega_c^2 + v_s^2 q^2}]$ as these correspond to bulk excitations. Hence, we must choose a contour within the spectral gap $0 < \omega < \min[\sqrt{\Omega_c^2 + v_s^2 q^2}]$, where ω is the photon energy. The dispersion is parabolic when $2\omega_c \nu_H < v_s^2$ and has a minimum at $q = 0$ with value $\min[\sqrt{\Omega_c^2 + v_s^2 q^2}] = \omega_c$. When $2\omega_c \nu_H > v_s^2$ however, the dispersion resembles a Mexican-hat potential and has a minimum at $q = \sqrt{(2\omega_c \nu_H - v_s^2)/2\nu_H^2}$ with value $\min[\sqrt{\Omega_c^2 + v_s^2 q^2}] = (v_s/2\nu_H)\sqrt{4\omega_c \nu_H - v_s^2}$. In graphene, the dispersion will generally be parabolic $2\omega_c \nu_H < v_s^2$ due to the large Fermi velocity. N is therefore defined within the spectral gap $0 < \omega < |\omega_c|$ where bulk current propagation is forbidden. We perform the integral over all imaginary frequencies $\Im(\Omega) \in (-i\infty, +i\infty)$,

$$\frac{-i}{2\pi^2} \int_{\omega - i\infty}^{\omega + i\infty} \frac{d\Omega}{\Omega^2} \frac{v_s^4 q^2 (\omega_c + \nu_H q^2)}{(\Omega^2 - \Omega_c^2 - v_s^2 q^2)^2} = -\text{res} \left[\frac{v_s^4 q^2}{\pi \Omega^2} \frac{\omega_c + \nu_H q^2}{(\Omega^2 - \Omega_c^2 - v_s^2 q^2)^2} \right]_{\Omega = \sqrt{\Omega_c^2 + v_s^2 q^2}} = \frac{3}{4\pi} \frac{v_s^4 q^2 (\omega_c + \nu_H q^2)}{(\Omega_c^2 + v_s^2 q^2)^{5/2}}, \tag{37}$$

and all wave vectors to obtain,

$$\boxed{N = \int q dq d\phi \frac{3}{4\pi} \frac{v_s^4 q^2 (\omega_c + \nu_H q^2)}{(\Omega_c^2 + v_s^2 q^2)^{5/2}} = \text{sgn}(\omega_c) + \text{sgn}(\nu_H)}. \tag{38}$$

$|N| = 2$ is nontrivial whenever $\omega_c \nu_H > 0$ and trivial $|N| = 0$ when $\omega_c \nu_H < 0$.

Supplementary Note 4: Fresnel equations of 2D electron fluids

For simplicity, we assume the surface charge at $z = 0$ is deposited on a dielectric substrate $n_-^2 = \varepsilon_-$ for $z < 0$ and a dielectric superstrate $n_+^2 = \varepsilon_+$ for $z > 0$. The wave vector in each of the dielectrics is $n_\pm \omega = ck_\pm = c\sqrt{q^2 + k_{z\pm}^2}$ and in-plane wave vector $\mathbf{q} \cdot \mathbf{q} = q^2$. The incident electric and magnetic fields can be expressed in an orthogonal $\hat{s}$ and $\hat{p}$ basis as,

$$\vec{\mathcal{E}}_0(z) = (\mathcal{E}_{0s} \hat{s} + \mathcal{E}_{0p} \hat{p}'_+) e^{-ik_{z+} z}, \qquad \vec{\mathcal{H}}_0(z) = n_+ (-\mathcal{E}_{0s} \hat{p}'_+ + \mathcal{E}_{0p} \hat{s}) e^{-ik_{z+} z}. \tag{39a}$$

The incident wave travels toward the interface at $z = 0$. The unit polarization vectors are defined as,

$$\hat{s} = \frac{1}{q} \begin{bmatrix} q_y \\ -q_x \\ 0 \end{bmatrix}, \qquad \hat{p}_+ = \frac{1}{k_+ q} \begin{bmatrix} -q_x k_{z+} \\ -q_y k_{z+} \\ q^2 \end{bmatrix}, \qquad \hat{p}'_+ = \hat{p}_+(-k_{z+}) = \frac{1}{k_+ q} \begin{bmatrix} q_x k_{z+} \\ q_y k_{z+} \\ q^2 \end{bmatrix}. \tag{39b}$$

$\hat{p}'$ is the unit vector of the reverse propagating $-k_z$ wave and has in-plane components $\hat{p}'_i = \hat{q}_i \zeta = -\hat{p}_i$. The in-plane components can be represented as $\hat{s}_i = \epsilon_{ij} \hat{q}^j$ and $\hat{p}_i = -\hat{q}_i \zeta$ which are orthogonal $\hat{s}_i \hat{p}^i = 0$. Moreover, defining the cosine $\zeta = k_z/k$, we have $\epsilon_{ij} \hat{s}^j = \zeta^{-1} \hat{p}_i$ and $\epsilon_{ij} \hat{p}^j = -\zeta \hat{s}_i$. The reflected field travels upward $+z$ away from the interface,

$$\vec{\mathcal{E}}_r(z) = [(\mathcal{E}_{0s} r_{ss} + \mathcal{E}_{0p} r_{sp}) \hat{s} + (\mathcal{E}_{0s} r_{ps} + \mathcal{E}_{0p} r_{pp}) \hat{p}_+] e^{ik_{z+} z}, \tag{40a}$$

while the reflected magnetic field is,

$$\vec{\mathcal{H}}_r(z) = n_+ [-(\mathcal{E}_{0s} r_{ss} + \mathcal{E}_{0p} r_{sp}) \hat{p}_+ + (\mathcal{E}_{0s} r_{ps} + \mathcal{E}_{0p} r_{pp}) \hat{s}] e^{ik_{z+} z}. \tag{40b}$$

Finally, the transmitted fields are,

$$\vec{\mathcal{E}}_t(z) = [(\mathcal{E}_{0s} t_{ss} + \mathcal{E}_{0p} t_{sp}) \hat{s} + (\mathcal{E}_{0s} t_{ps} + \mathcal{E}_{0p} t_{pp}) \hat{p}'_-] e^{-ik_{z-} z}, \tag{41a}$$

with,

$$\vec{\mathcal{H}}_t(z) = n_-[-(\mathcal{E}_{0s}t_{ss} + \mathcal{E}_{0p}t_{sp})\hat{p}'_- + (\mathcal{E}_{0s}t_{ps} + \mathcal{E}_{0p}t_{pp})\hat{s}]e^{-ik_{z-}z}, \tag{41b}$$

which propagates away from the interface $-z$.

First we utilize Supplementary Eq. (4c) to equate tangential electric fields E_i at $z = 0$ in the $\hat{s}_i$ and $\hat{q}_i$ directions,

$$\mathcal{E}_{0s} + \mathcal{E}_{0s}r_{ss} + \mathcal{E}_{0p}r_{sp} = \mathcal{E}_{0s}t_{ss} + \mathcal{E}_{0p}t_{sp}, \qquad \zeta_+(\mathcal{E}_{0p} - \mathcal{E}_{0s}r_{ps} - \mathcal{E}_{0p}r_{pp}) = \zeta_-(\mathcal{E}_{0s}t_{ps} + \mathcal{E}_{0p}t_{pp}), \tag{42}$$

which gives 4 equations relating the reflection and transmission coefficients,

$$\boxed{1 + r_{ss} = t_{ss}, \qquad r_{sp} = t_{sp}, \qquad \zeta_+(1 - r_{pp}) = \zeta_-t_{pp}, \qquad -\zeta_+r_{ps} = \zeta_-t_{ps}}. \tag{43}$$

Now we use Supplementary Eq. (4b) to relate the in-plane magnetic field to the surface charge. In the $\hat{s}_i$ and $\hat{q}_i$ basis we have,

$$\begin{aligned}\epsilon_{ij}\Delta\mathcal{H}^j &= (-n_+\zeta_+\mathcal{E}_{0s} + n_+\zeta_+\mathcal{E}_{rs} + n_-\zeta_-\mathcal{E}_{ts})\hat{s}_i + (-n_+\mathcal{E}_{0p} - n_+\mathcal{E}_{rp} + n_-\mathcal{E}_{tp})\hat{q}_i \\ = i4\pi\frac{\omega}{c}\chi_{ij}E^j &= i4\pi\frac{\omega}{c}[\chi^T(\mathcal{E}_{0s} + \mathcal{E}_{rs})\hat{s}_i + \zeta_+\chi^L(\mathcal{E}_{0p} - \mathcal{E}_{rp})\hat{q}_i - ig(\mathcal{E}_{0s} + \mathcal{E}_{rs})\hat{q}_i + ig\zeta_+(\mathcal{E}_{0p} - \mathcal{E}_{rp})\hat{s}_i].\end{aligned} \tag{44}$$

This gives us two equations for $\hat{s}$ and $\hat{q}$,

$$n_+\zeta_+(\mathcal{E}_{0s} - \mathcal{E}_{0s}r_{ss} - \mathcal{E}_{0p}r_{sp}) - n_-\zeta_-(\mathcal{E}_{0s}t_{ss} + \mathcal{E}_{0p}t_{sp}) = -i4\pi\frac{\omega}{c}[\chi^T(\mathcal{E}_{0s} + \mathcal{E}_{0s}r_{ss} + \mathcal{E}_{0p}r_{sp}) + ig\zeta_+(\mathcal{E}_{0p} - \mathcal{E}_{0s}r_{ps} - \mathcal{E}_{0p}r_{pp})] \tag{45a}$$

and,

$$n_+(\mathcal{E}_{0p} + \mathcal{E}_{0s}r_{ps} + \mathcal{E}_{0p}r_{pp}) - n_-(\mathcal{E}_{0s}t_{ps} + \mathcal{E}_{0p}t_{pp}) = -i4\pi\frac{\omega}{c}[\zeta_+\chi^L(\mathcal{E}_{0p} - \mathcal{E}_{0s}r_{ps} - \mathcal{E}_{0p}r_{pp}) - ig(\mathcal{E}_{0s} + \mathcal{E}_{0s}r_{ss} + \mathcal{E}_{0p}r_{sp})]. \tag{45b}$$

We can decompose this into 4 equations,

$$\boxed{n_+\zeta_+(1 - r_{ss}) - n_-\zeta_-t_{ss} = -i4\pi\frac{\omega}{c}[\chi^T(1 + r_{ss}) - i\zeta_+gr_{ps}]}, \tag{46a}$$

$$\boxed{n_+\zeta_+r_{sp} + n_-\zeta_-t_{sp} = i4\pi\frac{\omega}{c}[\chi^Tr_{sp} + i\zeta_+g(1 - r_{pp})]}, \tag{46b}$$

$$\boxed{n_+(1 + r_{pp}) - n_-t_{pp} = -i4\pi\frac{\omega}{c}[\zeta_+\chi^L(1 - r_{pp}) - igr_{sp}]}, \tag{46c}$$

$$\boxed{n_+r_{ps} - n_-t_{ps} = i4\pi\frac{\omega}{c}[\zeta_+\chi^Lr_{ps} + ig(1 + r_{ss})]}. \tag{46d}$$

We rearrange and isolate for the reflection coefficients. This can be expressed as a 2×2 matrix,

$$\begin{bmatrix}\mathcal{E}_{rs} \\ \mathcal{E}_{rp}\end{bmatrix} = \begin{bmatrix}r_{ss} & r_{sp} \\ r_{ps} & r_{pp}\end{bmatrix}\begin{bmatrix}\mathcal{E}_{0s} \\ \mathcal{E}_{0p}\end{bmatrix}. \tag{47}$$

The cross polarization r_{sp} and r_{ps} terms are known as the magneto-optic Kerr effect (MOKE). Noting that the cosine in each dielectric $\zeta_\pm = k_{z\pm}/k_\pm$ with $k_{z\pm} = \sqrt{k_\pm^2 - q^2}$ and $k_\pm = n_\pm\omega/c$ we have,

$$D^2 = (4\pi\omega/c)^2(\chi^T\chi^L - g^2), \tag{48a}$$

$$\boxed{r_{ss} = \frac{(n_+\zeta_+ - n_-\zeta_-)(n_+\zeta_+^{-1} + n_-\zeta_-^{-1}) + i4\pi(\omega/c)[(n_+\zeta_+^{-1} + n_-\zeta_-^{-1})\chi^T - (n_+\zeta_+ - n_-\zeta_-)\chi^L] + D^2}{(n_+\zeta_+ + n_-\zeta_-)(n_+\zeta_+^{-1} + n_-\zeta_-^{-1}) - i4\pi(\omega/c)[(n_+\zeta_+^{-1} + n_-\zeta_-^{-1})\chi^T + (n_+\zeta_+ + n_-\zeta_-)\chi^L] - D^2}}, \tag{48b}$$

$$\boxed{r_{ps} = r_{sp} = \frac{-2n_+4\pi(\omega/c)g}{(n_+\zeta_+ + n_-\zeta_-)(n_+\zeta_+^{-1} + n_-\zeta_-^{-1}) - i4\pi(\omega/c)[(n_+\zeta_+^{-1} + n_-\zeta_-^{-1})\chi^T + (n_+\zeta_+ + n_-\zeta_-)\chi^L] - D^2}}, \tag{48c}$$

$$\boxed{r_{pp} = -\frac{(n_+\zeta_+ + n_-\zeta_-)(n_+\zeta_+^{-1} - n_-\zeta_-^{-1}) + i4\pi(\omega/c)[(n_+\zeta_+ + n_-\zeta_-)\chi^L - (n_+\zeta_+^{-1} - n_-\zeta_-^{-1})\chi^T] + D^2}{(n_+\zeta_+ + n_-\zeta_-)(n_+\zeta_+^{-1} + n_-\zeta_-^{-1}) - i4\pi(\omega/c)[(n_+\zeta_+^{-1} + n_-\zeta_-^{-1})\chi^T + (n_+\zeta_+ + n_-\zeta_-)\chi^L] - D^2}}, \tag{48d}$$

The equations simplify significantly when there is reflection symmetry $n_+ = n_-$ but this is generally not true for the setups we are interested in. It is also straightforward to show that these reduce to the classical Fresnel equations when we remove the surface charge density. The MOKE rotation (s-effect) is defined by the ratio of reflected $\hat{p}$ to $\hat{s}$ polarization due to an incident $\hat{s}$ polarized wave,

$$\boxed{\tan\left(\frac{\Theta}{2}\right)\exp(i\Phi) = \frac{r_{ps}}{r_{ss}}}. \tag{49}$$

Since $\Theta \propto g$ is directly proportional to the gyrotropy g, any zero in $g = 0$ will result in a zero in the MOKE $\Theta = 0$.

Supplementary Note 5: Viscous bulk magnetoplasmons

Here we derive the dispersion of the bulk magnetoplasmon (BMP) and understand the influence of Hall viscosity ν_H on the bulk physics. These are the self-consistent field solutions of Maxwell's equations and the Navier-Stokes equations. We focus strictly on the quantum (dissipationless) limit $\Gamma \to 0$. Since the electronic oscillations are low frequency and extremely deep subwavelength $(v_s/c)^2 \ll 1$, we can utilize the quasistatic approximation $\vec{\nabla}\times\vec{\mathcal{E}} = \vec{0}$, also known as the Galilean approximation,

$$\vec{\mathcal{E}}(t,\vec{r}) = -\vec{\nabla}\phi(t,\vec{r}). \tag{50}$$

The electric field $\vec{\mathcal{E}}$ is essentially purely longitudinal. The electric potential ϕ is generated from fluctuations in the charge density ϱ and is evaluated through the divergence of the electric displacement field $\vec{\mathcal{D}}(t,\vec{r}) = -\varepsilon(z)\vec{\nabla}\phi(t,\vec{r})$. From this we construct Poisson's equation,

$$-\vec{\nabla}\cdot[\varepsilon(z)\vec{\nabla}\phi(t,\vec{r})] = 4\pi\delta(z)\varrho(t,\mathbf{r}). \tag{51}$$

$\varepsilon(z) = \varepsilon_+\Theta(z) + \varepsilon_-\Theta(-z)$ is the dielectric function in the regions above and below the Hall fluid, which captures the surface effects. $\Theta(z)$ being the Heaviside step function. For simplicity, we assume $\varepsilon_\pm \geq 1$ are positive scalar constants although dispersive and nonlocal effects can be included straightforwardly. The δ-function at $z = 0$ creates a discontinuity in the normal electric field $\mathcal{E}_z = -\partial_z\phi$. Integrating over the infinitesimal width of the 2D electron fluid yields,

$$-\varepsilon_+\frac{\partial\phi(t,\vec{r})}{\partial z}\bigg|_{z=0^+} + \varepsilon_-\frac{\partial\phi(t,\vec{r})}{\partial z}\bigg|_{z=0^-} = 4\pi\varrho(t,\mathbf{r}). \tag{52}$$

Although $\mathcal{E}_z$ is discontinuous, the potential must be continuous $\phi(t,\mathbf{r},0^-) = \phi(t,\mathbf{r},0^+)$ through the Hall fluid. Moreover, the potential satisfies Laplace's equation $\vec{\nabla}^2\phi(t,\vec{r}) = 0$ in the immediate vicinity of the 2D charge density and therefore decays as $|z| \to \infty$. This permits a simple representation in the momentum space.

Bulk dispersion relation

We consider an unbounded Hall fluid. Due to translational symmetry in the x-y plane, the in-plane momentum $\mathbf{q} = (q_x, q_y)$ is conserved. Hence, the potential ϕ can be expressed in the frequency-momentum space as,

$$\phi(t, \vec{r}) = \phi_{\mathbf{q}} e^{i\mathbf{q}\cdot\mathbf{r}} e^{-i\omega t} e^{-q|z|}, \tag{53}$$

where $q = \sqrt{\mathbf{q}\cdot\mathbf{q}}$ is the magnitude of the momentum. In the bulk momentum space, the potential $\phi_{\mathbf{q}}$ and fluctuating charge density $\varrho_{\mathbf{q}}$ are related by,

$$\varepsilon q \phi_{\mathbf{q}} = 2\pi \varrho_{\mathbf{q}}, \tag{54}$$

where $\varepsilon = (\varepsilon_+ + \varepsilon_-)/2$ is the average dielectric constant at the interface. Conversely, the presence of the potential generates current due to the force gradient $P_{i\mathbf{q}} = -i\chi_{ij} q^j \phi_{\mathbf{q}}$. The induced current density must simultaneously satisfy the continuity equation $\varrho_{\mathbf{q}} = -i\mathbf{q}\cdot\mathbf{P}_{\mathbf{q}}$, which imposes a self-consistency constraint. From this we obtain the characteristic equation,

$$\boxed{\varepsilon + 2\pi q \chi^L(\omega, q) = 0}. \tag{55}$$

Supplementary Equation (55) is only satisfied for certain vales of ω and q which establishes the dispersion relation $\omega = \omega_b(q)$ of the BMP. In the quantum limit $\Gamma \to 0$, the frequency of oscillations are purely real $\Im(\omega) = 0$. We obtain the BMP dispersion,

$$\boxed{\omega_b^2(q) = \Omega_p^2(q) + \Omega_c^2(q) + v_s^2 q^2}, \tag{56}$$

where,

$$\Omega_p(q) = \sqrt{\frac{2\pi e^2 n_0 q}{m\varepsilon}}, \qquad \Omega_c(q) = \omega_c - \nu_H q^2. \tag{57}$$

$\Omega_p(q)$ is the effective plasma frequency and is momentum dependent in 2D. $\Omega_c(q)$ is the effective cyclotron frequency which is momentum dependent due to Hall viscosity ν_H. As expected, the biasing magnetic field $B \neq 0$ creates a low frequency band gap at $q = 0$, precisely at the cyclotron frequency $\omega_b(0) = |\omega_c|$. We also recover the square root dispersion $\omega_b(q) \propto \sqrt{q}$ for low momenta $q \approx 0$ which is characteristic of surface plasmons. However, for ultra high momenta $q \to \infty$, the BMP dispersion is parabolic $\omega_b(q) \propto q^2$ since viscosity dominates in the deep wavelength. This has multiple important consequences. Topologically, it regularizes the continuum theory at $q \to \infty$ by introducing a mesoscopic length scale $D_H = \sqrt{\nu_H/\omega_c}$.

Dynamical variables of the viscous Hall fluid: energy and angular momentum

In this section, we derive the necessary quantities to understand the energy and angular momentum of the BMP in the quasistatic regime. The momentum density $\vec{\mathcal{S}}$ is derived from the Poynting vector as,

$$\vec{\mathcal{S}}(t, \vec{r}) = \frac{c}{4\pi} \vec{\mathcal{E}}(t, \vec{r}) \times \vec{\mathcal{H}}(t, \vec{r}). \tag{58}$$

The divergence of $\vec{\mathcal{S}}$ gives Poynting's theorem in the quasistatic limit,

$$\vec{\nabla}\cdot\vec{\mathcal{S}}(t, \vec{r}) + \dot{u}(t, \vec{r}) = -\vec{\mathcal{E}}(t, \vec{r})\cdot\vec{\mathcal{J}}(t, \vec{r}), \qquad \dot{u}(t, \vec{r}) = \frac{1}{4\pi}\vec{\mathcal{E}}(t, \vec{r})\cdot\frac{\partial}{\partial t}\vec{\mathcal{D}}(t, \vec{r}). \tag{59}$$

As we can see, only the electric field contributes to the energy density u as the magnetic field contributions are negligible. Note that,

$$\boxed{\dot{U}(t) = \int d\vec{r}\, \dot{u}(t,\vec{r}) = \frac{1}{4\pi}\int d\vec{r}\, \vec{\mathcal{E}}(t,\vec{r})\cdot\frac{\partial}{\partial t}\vec{\mathcal{D}}(t,\vec{r}) = \int d\mathbf{r}\ \phi(t,\mathbf{r})\frac{\partial}{\partial t}\varrho(t,\mathbf{r})} \tag{60}$$

where we have integrated by parts and used $\vec{\nabla}\cdot\vec{\mathcal{D}}(t,\vec{r}) = 4\pi\delta(z)\varrho(t,\mathbf{r})$. We follow the conventional procedure for deriving u with dispersion. The 2D charge density is related to ϕ through the susceptibility tensor,

$$\begin{aligned}\varrho(t,\mathbf{r}) &= \int_{-\infty}^{t} dt' \int d\mathbf{r}' \nabla^i \chi_{ij}(t-t',\mathbf{r},\mathbf{r}')\nabla^{j'}\phi(t',\mathbf{r}') = \int_{-\infty}^{t} dt' \int d\mathbf{r}' X(t-t',\mathbf{r},\mathbf{r}')\phi(t',\mathbf{r}')\\ &= \int_{0}^{\infty} dt' \int d\mathbf{r}' X(t',\mathbf{r},\mathbf{r}')\phi(t-t',\mathbf{r}'),\end{aligned} \tag{61}$$

where we have integrated by parts,

$$X(t,\mathbf{r},\mathbf{r}') = -\nabla^i\nabla^{j'}\chi_{ij}(t,\mathbf{r},\mathbf{r}'). \tag{62}$$

Now consider an arbitrary eigenmode of the system $\phi_n(t,\vec{r}) = [\phi_n(\vec{r})e^{-i\omega_n t} + \phi_n^*(\vec{r})e^{i\omega_n t}]/\sqrt{2}$. The scalar product with another arbitrary mode n' is $U_{n'n} = \int_0^\infty dt\dot{U}_{n'n}(t)$,

$$\begin{aligned}U_{n'n} &= \frac{1}{2}\iint d\mathbf{r}d\mathbf{r}' \int_0^\infty dt \int_0^\infty dt' [\phi_{n'}(\mathbf{r})e^{-i\omega_{n'}t} + \phi_{n'}^*(\mathbf{r})e^{i\omega_{n'}t}]X(t',\mathbf{r},\mathbf{r}')\partial_t[\phi_n(\mathbf{r}')e^{-i\omega_n(t-t')} + \phi_n^*(\mathbf{r}')e^{i\omega_n(t-t')}]\\ &= \frac{-i}{2}\iint d\mathbf{r}d\mathbf{r}' \int_0^\infty dt [\phi_{n'}(\mathbf{r})e^{-i\omega_{n'}t} + \phi_{n'}^*(\mathbf{r})e^{i\omega_{n'}t}][\omega_n X(\omega_n,\mathbf{r},\mathbf{r}')\phi_n(\mathbf{r}')e^{-i\omega_n t} - \omega_n X(-\omega_n,\mathbf{r},\mathbf{r}')\phi_n^*(\mathbf{r}')e^{i\omega_n t}]\\ &= \frac{1}{2}\iint d\mathbf{r}d\mathbf{r}'\left[\phi_{n'}^*(\mathbf{r})\frac{\omega_n X(\omega_n,\mathbf{r},\mathbf{r}')}{\omega_{n'}-\omega_n}\phi_n(\mathbf{r}') + \phi_{n'}(\mathbf{r})\frac{\omega_n X^*(\omega_n,\mathbf{r},\mathbf{r}')}{\omega_{n'}-\omega_n}\phi_n^*(\mathbf{r}')\right]\\ &= \frac{1}{2}\iint d\mathbf{r}d\mathbf{r}'\left[\phi_{n'}^*(\mathbf{r})\frac{\omega_n X(\omega_n,\mathbf{r},\mathbf{r}')}{\omega_{n'}-\omega_n}\phi_n(\mathbf{r}') + \phi_n^*(\mathbf{r})\frac{\omega_n X(\omega_n,\mathbf{r},\mathbf{r}')}{\omega_{n'}-\omega_n}\phi_{n'}(\mathbf{r}')\right].\end{aligned} \tag{63a}$$

But $U_{n'n}^* = U_{n'n} = U_{nn'}$ is real so,

$$\phi_n^*(\mathbf{r})\frac{\omega_n X(\omega_n,\mathbf{r},\mathbf{r}')}{\omega_{n'}-\omega_n}\phi_{n'}(\mathbf{r}') = \phi_{n'}^*(\mathbf{r})\frac{\omega_{n'} X(\omega_{n'},\mathbf{r},\mathbf{r}')}{\omega_n-\omega_{n'}}\phi_n(\mathbf{r}'), \tag{63b}$$

and therefore,

$$U_{n'n} = -\frac{1}{2}\iint d\mathbf{r}d\mathbf{r}'\phi_{n'}^*(\mathbf{r})\frac{\omega_n X(\omega_n,\mathbf{r},\mathbf{r}') - \omega_{n'}X(\omega_{n'},\mathbf{r},\mathbf{r}')}{\omega_n-\omega_{n'}}\phi_n(\mathbf{r}'). \tag{63c}$$

Taking the limit of $n'\to n$

$$U_{nn} = -\frac{1}{2}\iint d\mathbf{r}d\mathbf{r}'\phi_n^*(\mathbf{r})\frac{\partial}{\partial\omega}\left[\omega X(\omega,\mathbf{r},\mathbf{r}')\right]_{\omega=\omega_n}\phi_n(\mathbf{r}'). \tag{64}$$

Assuming translational symmetry in the x-y plane $X(\omega,\mathbf{r},\mathbf{r}') = X(\omega,\mathbf{r}-\mathbf{r}')$, we can convert to the momentum space,

$$U_{nn} = -\frac{1}{2}\int\frac{d\mathbf{q}}{(2\pi)^2}\frac{\partial}{\partial\omega}\left[\omega X(\omega,\mathbf{q})\right]_{\omega=\omega_n}|\phi_{n\mathbf{q}}|^2. \tag{65}$$

Finally, assuming rotational symmetry in the x-y plane $X(\omega,\mathbf{q}) = -q^i q^j\chi_{ij}(\omega,\mathbf{q}) = -\chi^L(\omega,q)q^2$ we obtain,

$$\boxed{U = \int\frac{d\mathbf{q}}{(2\pi)^2}u_{\mathbf{q}},\qquad u_{\mathbf{q}} = \frac{1}{2}\frac{\partial}{\partial\omega}\left[\omega\chi^L(\omega,q)\right]q^2|\phi_{\mathbf{q}}|^2}, \tag{66}$$

for an arbitrary mode. Evaluating specifically for the BMP $\omega = \omega_b(q)$ we obtain,

$$\boxed{u_{\mathbf{q}} = \frac{m^2\varepsilon^2}{32\pi^3 e^4 n_0^2}\left[2\omega_b^2(q) - \Omega_p^2(q)\right]|\phi_{\mathbf{q}}|^2}. \tag{67}$$

Now we conduct the same procedure for the angular momentum. Due to rotational symmetry in the x-y plane, the relevant quantity is the angular momentum along $\hat{z}$. The torque density along $\hat{z}$ is expressed as,

$$\tau(t,\vec{r}) = \frac{1}{4\pi}\epsilon_{ij}\mathcal{E}^i(t,\vec{r})\mathcal{D}^j(t,\vec{r}), \tag{68}$$

which gives rate of change of the angular momentum M,

$$\boxed{\dot{M}(t) = \int d\vec{r}\,\tau(t,\vec{r}) = \frac{\epsilon_{ij}}{4\pi}\int d\vec{r}\,\mathcal{E}^i(t,\vec{r})\mathcal{D}^j(t,\vec{r}) = \int d\mathbf{r}\,\phi(t,\mathbf{r})\epsilon_{ij}\nabla^i P^j(t,\mathbf{r})} \tag{69}$$

We have integrated by parts and used $\vec{\mathcal{D}}(t,\vec{r}) = \vec{\mathcal{E}}(t,\vec{r}) + 4\pi\delta(z)\mathbf{P}(t,\mathbf{r})$. The form of this equation is very similar to the energy density and is evaluated in the same way. We note that,

$$\begin{aligned}\epsilon_{ij}\nabla^i P^j(t,\mathbf{r}) &= -\epsilon_{ij}\int_{-\infty}^{t} dt'\int d\mathbf{r}'\nabla^i\chi_{jk}(t-t',\mathbf{r},\mathbf{r}')\nabla^{k'}\phi(t',\mathbf{r}') = \int_{-\infty}^{t} dt'\int d\mathbf{r}' G(t-t',\mathbf{r},\mathbf{r}')\phi(t',\mathbf{r}')\\ &= \int_0^{\infty} dt'\int d\mathbf{r}' G(t',\mathbf{r},\mathbf{r}')\phi(t-t',\mathbf{r}'),\end{aligned} \tag{70}$$

where we have integrated by parts,

$$G(t,\mathbf{r},\mathbf{r}') = \epsilon_{ij}\nabla^i\nabla^{k'}\chi_{jk}(t,\mathbf{r},\mathbf{r}'). \tag{71}$$

Note that G is a pseudo-scalar and is odd under $\mathbf{r}\leftrightarrow\mathbf{r}'$. Now we analyze the overlap of two distinct modes. The scalar product is $M_{n'n} = \int_0^\infty \dot{M}_{n'n}(t)$,

$$\begin{aligned}M_{n'n} &= \frac{i}{2}\iint d\mathbf{r}d\mathbf{r}'\left[\phi_{n'}^*(\mathbf{r})\frac{G(\omega_n,\mathbf{r},\mathbf{r}')}{\omega_{n'}-\omega_n}\phi_n(\mathbf{r}') - \phi_{n'}(\mathbf{r}')\frac{G(-\omega_n,\mathbf{r},\mathbf{r}')}{\omega_{n'}-\omega_n}\phi_n^*(\mathbf{r}')\right]\\ &= \frac{i}{2}\iint d\mathbf{r}d\mathbf{r}'\left[\phi_{n'}^*(\mathbf{r})\frac{G(\omega_n,\mathbf{r},\mathbf{r}')}{\omega_{n'}-\omega_n}\phi_n(\mathbf{r}') + \phi_n^*(\mathbf{r})\frac{G(\omega_n,\mathbf{r},\mathbf{r}')}{\omega_{n'}-\omega_n}\phi_{n'}(\mathbf{r}')\right]\end{aligned} \tag{72a}$$

Again, $M_{n'n}^* = M_{n'n} = M_{nn'}$ is real so,

$$\phi_n^*(\mathbf{r})\frac{G(\omega_n,\mathbf{r},\mathbf{r}')}{\omega_{n'}-\omega_n}\phi_{n'}(\mathbf{r}') = \phi_{n'}^*(\mathbf{r})\frac{G(\omega_{n'},\mathbf{r},\mathbf{r}')}{\omega_n-\omega_{n'}}\phi_n(\mathbf{r}') \tag{72b}$$

and therefore,

$$M_{n'n} = -\frac{i}{2}\iint d\mathbf{r}d\mathbf{r}'\phi_{n'}^*(\mathbf{r})\frac{G(\omega_n,\mathbf{r},\mathbf{r}') - G(\omega_{n'},\mathbf{r},\mathbf{r}')}{\omega_n-\omega_{n'}}\phi_n(\mathbf{r}') \tag{72c}$$

Taking the limit of $n'\to n$

$$M_{nn} = -\frac{i}{2}\iint d\mathbf{r}d\mathbf{r}'\phi_n^*(\mathbf{r})\frac{\partial}{\partial\omega}\left[G(\omega,\mathbf{r},\mathbf{r}')\right]_{\omega=\omega_n}\phi_n(\mathbf{r}'). \tag{73}$$

Assuming translational symmetry in the x-y plane $G(\omega,\mathbf{r},\mathbf{r}') = G(\omega,\mathbf{r}-\mathbf{r}')$, we can convert to the momentum space,

$$M_{nn} = -\frac{i}{2}\int\frac{d\mathbf{q}}{(2\pi)^2}\frac{\partial}{\partial\omega}\left[G(\omega,\mathbf{q})\right]_{\omega=\omega_n}|\phi_{n\mathbf{q}}|^2. \tag{74}$$

Finally, with rotational symmetry in the x-y plane, $G(\omega, \mathbf{q}) = q^i q^k \epsilon_{ij} \chi_{jk} = -ig(\omega, k) q^2$ we obtain,

$$\boxed{M = \int \frac{d\mathbf{q}}{(2\pi)^2} m_{\mathbf{q}}, \qquad m_{\mathbf{q}} = -\frac{1}{2} \frac{\partial g(\omega, q)}{\partial \omega} q^2 |\phi_{\mathbf{q}}|^2}, \tag{75}$$

for an arbitrary mode. Evaluating specifically for the BMP $\omega = \omega_b(q)$ we obtain,

$$\boxed{m_{\mathbf{q}} = \frac{m^2 \varepsilon^2}{32\pi^3 e^4 n_0^2} \frac{\Omega_c(q)}{\omega_b^2(q)} \left[2\omega_b^2(q) + \Omega_p^2(q)\right] |\phi_{\mathbf{q}}|^2}. \tag{76}$$

Note that the angular momentum j_z per unit energy ω is defined as,

$$\frac{m_{\mathbf{q}}}{u_{\mathbf{q}}} = -\frac{\partial g}{\partial \omega} \Big/ \frac{\partial (\omega \chi^L)}{\partial \omega} = \frac{j_z}{\omega} \tag{77}$$

which can be evaluated for any particular mode For the viscous BMP specifically $\omega = \omega_b$ we have,

$$\frac{m_{\mathbf{q}}}{u_{\mathbf{q}}} = -\left[\frac{\partial g}{\partial \omega} \Big/ \frac{\partial (\omega \chi^L)}{\partial \omega}\right]_{\omega = \omega_b} = \frac{\Omega_c}{\omega_b^2} \left(\frac{2\omega_b^2 + \Omega_p^2}{2\omega_b^2 - \Omega_p^2}\right), \tag{78}$$

which gives an angular momentum j_z of,

$$\boxed{j_z = \frac{\Omega_c}{\omega_b} \left(\frac{2\omega_b^2 + \Omega_p^2}{2\omega_b^2 - \Omega_p^2}\right)}. \tag{79}$$

Note that due to rotational symmetry, the high-symmetry points $q = 0$ and $q = \infty$ must be integers $j_z = \pm 1, 0$ since the system is properly regularized. For the viscous BMP we have $j_z(0) = \mathrm{sgn}[\Omega_c(0)] = \mathrm{sgn}(\omega_c)$ and $j_z(\infty) = \mathrm{sgn}[\Omega_c(\infty)] = -\mathrm{sgn}(\nu_H)$. The difference is precisely the N-invariant $\Delta j_z = j_z(0) - j_z(\infty) = \mathrm{sgn}(\omega_c) + \mathrm{sgn}(\nu_H) = N$.

Supplementary Note 6: Topological edge magnetoplasmons

Here we derive the topological edge states of the dissipationless semi-infinite viscous Hall fluid – these are topological edge magnetoplasmons (EMPs). To accurately model the edge physics we need to impose appropriate boundary conditions on the fields. The electric potential ϕ and its normal derivative $\hat{n} \cdot \boldsymbol{\nabla} \phi$ are continuous across the boundary. However, due to nonlocality in a hydrodynamic electron fluid, we require additional boundary conditions (ABCs) on the current density $\mathbf{J}$. Charge conservation necessitates a vanishing normal current,

$$\boxed{\hat{n} \cdot \mathbf{J}|_{\partial V} = 0,} \tag{80a}$$

where $\hat{n}$ is the outward normal unit vector and ∂V denotes the boundary of some volume V. A viscous hydrodynamic fluid also requires a BC on the tangential current which is related to momentum conservation. The tangential current is proportional to the shear stress (off-diagonal stress) on ∂V [10],

$$\boxed{\left[-e\, \hat{t} \cdot \varsigma \cdot \hat{n} + m v_b \hat{t} \cdot \mathbf{J}\right]_{\partial V} = 0}. \tag{80b}$$

$\hat{t} = \hat{n} \times \hat{z}$ is the unit tangential vector, ς is the viscous stress tensor and v_b is the boundary scattering velocity that dictates the slip flow.

In the case of a half-space, Poisson's equation is modified to,

$$-\vec{\nabla} \cdot [\varepsilon(z) \vec{\nabla} \phi(t, \vec{r})] = 4\pi \delta(z) \Theta(x) \rho(t, \mathbf{r}). \tag{81}$$

$\varepsilon(z) = \varepsilon_+\Theta(z) + \varepsilon_-\Theta(-z)$ is the dielectric function in the regions above and below the Hall fluid, which captures the surface effects. $\Theta(z)$ being the Heaviside step function. The δ-function at $z = 0$ creates a discontinuity in the normal electric field $\mathcal{E}_z = -\partial_z\phi$. Integrating over the infinitesimal width of the 2D electron fluid yields,

$$-\varepsilon_+ \frac{\partial\phi(t,\vec{r})}{\partial z}\bigg|_{z=0^+} + \varepsilon_- \frac{\partial\phi(t,\vec{r})}{\partial z}\bigg|_{z=0^-} = 4\pi\Theta(x)\rho(t,\mathbf{r}). \tag{82}$$

Although $\mathcal{E}_z$ is discontinuous, the potential must be continuous $\phi(t,\mathbf{r},0^-) = \phi(t,\mathbf{r},0^+)$ through the Hall fluid. Moreover, the potential satisfies Laplace's equation $\vec{\nabla}^2\phi(t,\vec{r}) = 0$ in the immediate vicinity of the 2D charge density $z \neq 0$ and therefore decays as $|z| \to \infty$. This permits a simple representation in the momentum space. Due to translational symmetry along y, the transverse momentum q_y and frequency ω are still good quantum numbers. We Fourier transform over x to obtain,

$$\phi_k(z) = \phi_k e^{-|z|\sqrt{k^2+q_y^2}}, \tag{83a}$$

where k is the Fourier variable of x and $\phi_k = \phi_k(0)$ is related to the charge density by,

$$\phi_k = \frac{4\pi}{\varepsilon}\bar{L}_k\rho_k, \qquad \bar{L}_k = \frac{1}{2\sqrt{k^2+q_y^2}}, \qquad \rho_k = \int_0^\infty \rho(x)e^{-ikx}dx. \tag{83b}$$

$\varepsilon = (\varepsilon_+ + \varepsilon_-)/2$ is the average dielectric constant at the interface and $\bar{L}_k$ is the Poisson kernel. Transforming back to real space gives the potential at all points in the plane $z = 0$,

$$\phi(x) = \frac{1}{2\pi}\int_{-\infty}^{\infty}\phi_k e^{ikx}dk = \frac{4\pi}{\varepsilon}\int_0^\infty L(x-x')\rho(x')dx', \tag{84a}$$

where $L(x)$ is the Green's function (inverse transform) of $\bar{L}_k$,

$$L(x) = \frac{1}{2\pi}\int_{-\infty}^{\infty}\bar{L}_k e^{ikx}dk = \frac{1}{2\pi}K_0(|q_y x|). \tag{84b}$$

K_0 is the zeroth order modified Bessel function of the second kind. Note that the Green's function $L(x)$ diverges logarithmically $L(x) \sim -\log(|q_y x|/2)$ as the argument $|q_y x| \to 0$.

Fetter approximation

Unfortunately, the Green's function $L(x)$ does not satisfy a simple differential equation which can relate $\phi(x)$ and $\rho(x)$ locally. Due to the fringing fields in the surrounding dielectrics $z \neq 0$, we only possess a nonlocal integral relation which significantly complicates the problem. Problems of this type can always be solved via the Wiener-Hopf method [27]. However, due to the mathematical complexity of this technique, some of the intuition regarding the fundamental physics is lost. Instead we implement the Fetter approximation [28] which substitutes the Poisson kernel $\bar{L}_k$ with its paraxial approximation,

$$\bar{L}_k \approx \frac{|q_y|}{k^2+2q_y^2}, \qquad L(x) \approx \frac{e^{-\sqrt{2}|q_y x|}}{2\sqrt{2}}. \tag{85}$$

Note that the paraxial and exact kernels have the same first two terms in power series around $k^2 = 0$ but the paraxial function has a much simpler analytic structure as it is meromorphic. Indeed, the approximate Green's function satisfies a simple differential equation,

$$(2q_y^2 - \partial_x^2)L(x) = |q_y|\delta(x), \tag{86}$$

and therefore has a local representation. In the Fetter approximation we can break up the equation into two local half-spaces for $x \geq 0$ and $x < 0$,

$$\boxed{\varepsilon(2q_y^2 - \partial_x^2)\phi(x) = \begin{cases} 0 & x < 0 \\ 4\pi|q_y|\rho(x) & x \geq 0. \end{cases}} \tag{87}$$

The Fetter approximation is very accurate for moderate momentum q_y but produces a qualitatively different solution at $q_y \to 0$ compared to the full Wiener-Hopf method. The Fetter approximation generates a finite group velocity $\partial\omega/\partial q_y$ at $q_y \to 0$ and does not capture the true logarithmic divergence $\partial\omega/\partial q_y \to \infty$ due to the long-range Coulomb interaction [29]. However, this is not particularly important for the topological physics so we continue with the Fetter approach here. Note that the numerical simulations of the quantum circulator in the main manuscript use the full Coulomb potential to.

Boundary conditions and characteristic equation

For clarity, we label the potential in the two half-spaces as $\phi_-(x) = \phi(x < 0)$ and $\phi_+(x) = \phi(x \geq 0)$. The solution for ϕ_- is obvious. ϕ_+ is more complicated however because we have three independent decay vectors for arbitrary ω and q_y. Generically, the $\phi_\pm$ potentials can be expanded as a linear sum in decay constants η,

$$\phi_-(x) = \phi_0 e^{\sqrt{2}|q_y|x}, \qquad \phi_+(x) = \sum_{i=1}^{3} \phi_i e^{-\eta_i x}, \tag{88}$$

where the coefficients ϕ_i and edge dispersion $\omega = \omega_e(q_y)$ are to be determined by the boundary conditions. We also require $\Re\eta > 0$ to ensure the potential is evanescent and satisfies the boundary condition at infinity $\phi_+(\infty) = 0$. Inserting into the hydrodynamic Navier-Stokes equations [Supplementary Eq. (17)] we obtain the cubic dispersion relation for $q^2 = -\eta^2 + q_y^2$,

$$(q^2 + q_y^2)\left[\omega^2 - \Omega_c^2(q) - v_s^2 q^2\right] - 2\Omega_p^2 q^2 = 0, \qquad \Omega_p = \sqrt{\frac{2\pi e^2 n_0 |q_y|}{m\varepsilon}}, \tag{89a}$$

where Ω_p is the bulk 2D plasma frequency and $\Omega_c(q) = \omega_c - \nu_H q^2$. Expanding in powers of q^2 we obtain,

$$\boxed{-\nu_H^2 q^6 + (2\omega_c\nu_H - v_s^2 - \nu_H^2 q_y^2)q^4 + (\omega^2 - \omega_b^2 - \Omega_p^2 + \nu_H^2 q_y^4)q^2 + q_y^2(\omega^2 - \omega_c^2) = 0}. \tag{89b}$$

$\omega_b^2(q_y) = \Omega_p^2(q_y) + v_s^2 q_y^2 + \Omega_c^2(q_y)$ is the bulk plasmon dispersion. The dispersion relations gives 3 possible roots q_i^2 and therefore three decay constants $\eta_i = \sqrt{q_y^2 - q_i^2}$. The induced current for $x \geq 0$ is then,

$$\delta\rho(x) = \frac{e^2 n_0}{2m\Omega_p^2} \sum_{i=1}^{3} (q_i^2 + q_y^2)\phi_i e^{-\eta_i x}, \tag{90a}$$

$$J_x(x) = i\frac{e^2 n_0}{2m\Omega_p^2} \sum_{i=1}^{3} (\omega\eta_i - \Omega_{ci} q_y)\frac{q_i^2 + q_y^2}{q_i^2}\phi_i e^{-\eta_i x}, \tag{90b}$$

$$J_y(x) = \frac{e^2 n_0}{2m\Omega_p^2} \sum_{i=1}^{3} (\omega q_y - \Omega_{ci}\eta_i)\frac{q_i^2 + q_y^2}{q_i^2}\phi_i e^{-\eta_i x}, \tag{90c}$$

where $\Omega_{ci} = \omega_c - \nu_H q_i^2$.

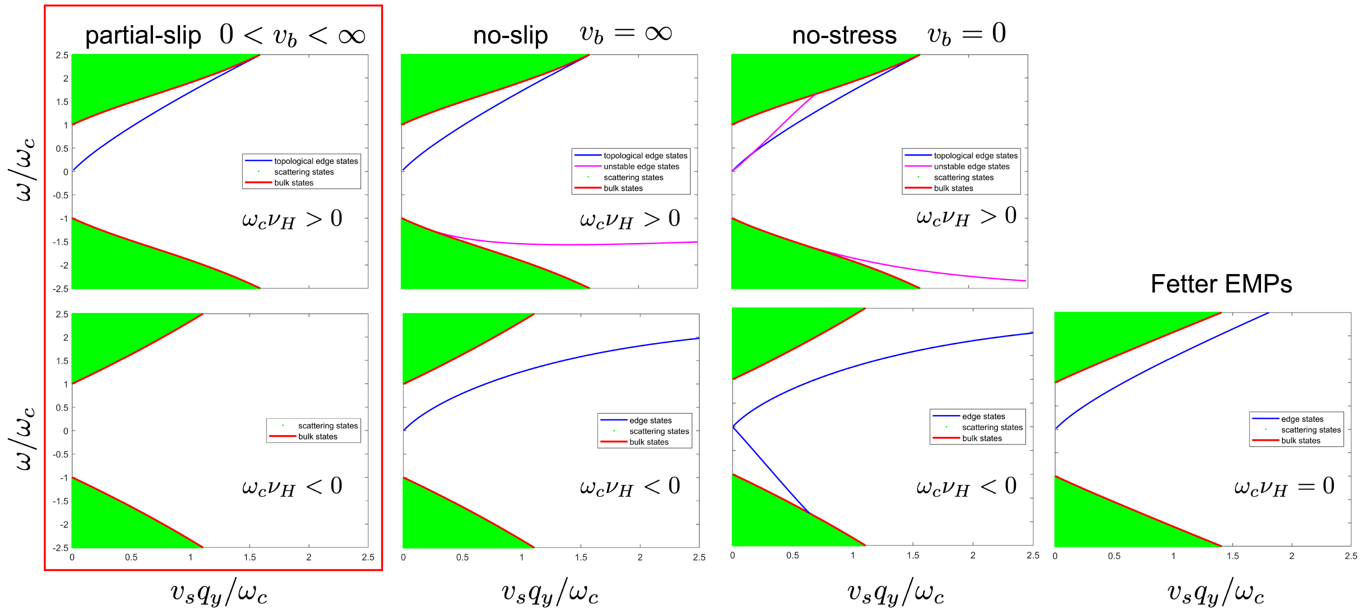

Supplementary Fig. 1: **Edge states for partial-slip, no-slip and stress-free boundary conditions in nontrivial and trivial regimes.** As we can see, the topological edge state (blue curve in top panels) is the only stable solution that exists for all possible boundary conditions. All other edge states are unstable as they can be gapped out by continuously deforming the boundary condition $0 < v_b < \infty$. Fetter EMPs with $\nu_H = 0$ are not regularized and gapped in the hydrodynamic continuum theory and therefore do not possess a topological interpretation.

We now implement the boundary conditions on $x = 0$. We have four unknowns which requires the specification of four boundary conditions. The continuity of the potential $\phi_+(0) = \phi_-(0)$ follows immediately and requires,

$$\phi_0 = \sum_{i=1}^{3} \phi_i. \tag{91}$$

We also require continuity of the normal derivative $\partial_x \phi_+(0) = \partial_x \phi_-(0)$. We confirm this by integrating the Fetter equation [Supplementary Eq. (87)] over an infinitesimal width,

$$-\partial_x \phi(x)\Big|_{0^-}^{0^+} = \int_0^{0^+} \rho(x) dx = 0. \tag{92}$$

Since ρ contains no δ-functions, the integral vanishes. This produces a second relation,

$$\sqrt{2}|q_y|\phi_0 + \sum_{i=1}^{3} \eta_i \phi_i = 0. \tag{93}$$

We must also impose boundary conditions on the current density $\mathbf{J}$. Charge conservation demands the vanishing of $J_x(0) = 0$ on the boundary $x = 0$,

$$\sum_{i=1}^{3} (\omega \eta_i - \Omega_{ci} q_y) \frac{q_i^2 + q_y^2}{q_i^2} \phi_i = 0. \tag{94}$$

For a viscous hydrodynamic fluid, we require a boundary condition on the tangential current J_y which ensures momentum conservation. In the quantum limit, with outward normal vector $\hat{n} = -\hat{x}$ and tangential vector $\hat{t} = \hat{y}$, the boundary condition requires that the tangential stress ς_{xy} be proportional to the tangential current J_y. This is a statement of momentum conservation,

$$l_H(\partial_x J_x - i q_y J_y)|_{x=0} = J_y|_{x=0}, \tag{95}$$

where $l_H = \nu_H/v_b$ is the Hall slip length and $v_b \geq 0$ is the boundary scattering velocity. Using the continuity equation, this can be expressed as,

$$[\omega l_H \rho + (1 + 2iq_y l_H) J_y]_{x=0} = 0. \tag{96}$$

Note that l_H is either positive or negative depending on the sign of ν_H. The two extremes are the no-slip $|l_H| = 0$ and stress-free $|l_H| = \infty$ boundary conditions, while the partial-slip $|l_H| \neq 0$ is a mixed boundary condition. Generically, the mixed boundary condition on J_y with $|l_H| \neq 0$ finite can be expressed as,

$$\sum_{i=1}^{3} \left\{ i l_H [2q_y(\omega q_y - \Omega_{ci}\eta_i) - \omega q_i^2] + \omega q_y - \Omega_{ci}\eta_i \right\} \frac{q_i^2 + q_y^2}{q_i^2} \phi_i = 0. \tag{97}$$

Combining this with the previous set of equations generates the edge dispersion $\omega = \omega_e(q_y)$. It would appear that the value of l_H can affect the dispersion, which it will for spurious unstable solutions. However, if no-slip and stress-free are satisfied simultaneously, then the edge wave is independent of l_H,

$$J_y|_{x=0} = (\partial_x J_x - \partial_y J_y)|_{x=0} = \partial_t \rho|_{x=0} = 0. \tag{98}$$

Note that no-slip and stress-free together imply $\partial_t \rho$ vanishes. Hence, we get two additional constraints on the current density,

$$\sum_{i=1}^{3} (\omega q_y - \Omega_{ci}\eta_i) \frac{q_i^2 + q_y^2}{q_i^2} \phi_i = 0, \qquad \sum_{i=1}^{3} (q_i^2 + q_y^2) \phi_i = 0, \tag{99}$$

which must be combined with the BCs on ϕ. In Supplementary Fig. 1 we solve for the edge states in the no-slip, stress-free and partial-slip boundary conditions. As we can see, only the topologically protected edge state in the nontrivial regime $\omega_c \nu_H > 0$ persist for arbitrary values of $l_H = \nu_H/v_b$. The spurious solutions in the no-slip and stress-free cases can be gapped out for any finite values of $l_H = \nu_H/v_b$ and are therefore unstable. Notice that the edge magnetoplasmons predicted by Fetter (with $\nu_H = 0$) do not possess a topological interpretation and are not stable as they can be gapped out by perturbations.

Ultra-subwavelength topological circulators

We exploit the robust chiral edge plasmons in the nontrivial phase $N = 2$ to design an ultra-subwavelength topological circulator. Circulators are passive 3- or 4-port devices that route signals in specific directions and are considered the fundamental nonreciprocal component since both isolators and gyrators can be constructed from them. Signals that enter a port are transmitted solely to the next adjacent port in either a clockwise or counterclockwise fashion with no reflection. We consider a 3-port geometry as pictured in Supplementary Fig. 2. Dipoles are placed near the entry ports and oscillate at a frequency in the band gap. The signals are transmitted clockwise sequentially from $1 \to 2 \to 3 \to 1$ with no back-scattering and navigate sharp defects with impunity.

---

[1] J. D. Jackson, *Classical electrodynamics* (John Wiley & Sons, 2007).

[2] D. A. Bandurin, I. Torre, R. K. Kumar, M. Ben Shalom, A. Tomadin, A. Principi, G. H. Auton, E. Khestanova, K. S. Novoselov, I. V. Grigorieva, et al., Science **351**, 1055 (2016), ISSN 0036-8075, URL `https://science.sciencemag.org/content/351/6277/1055`.

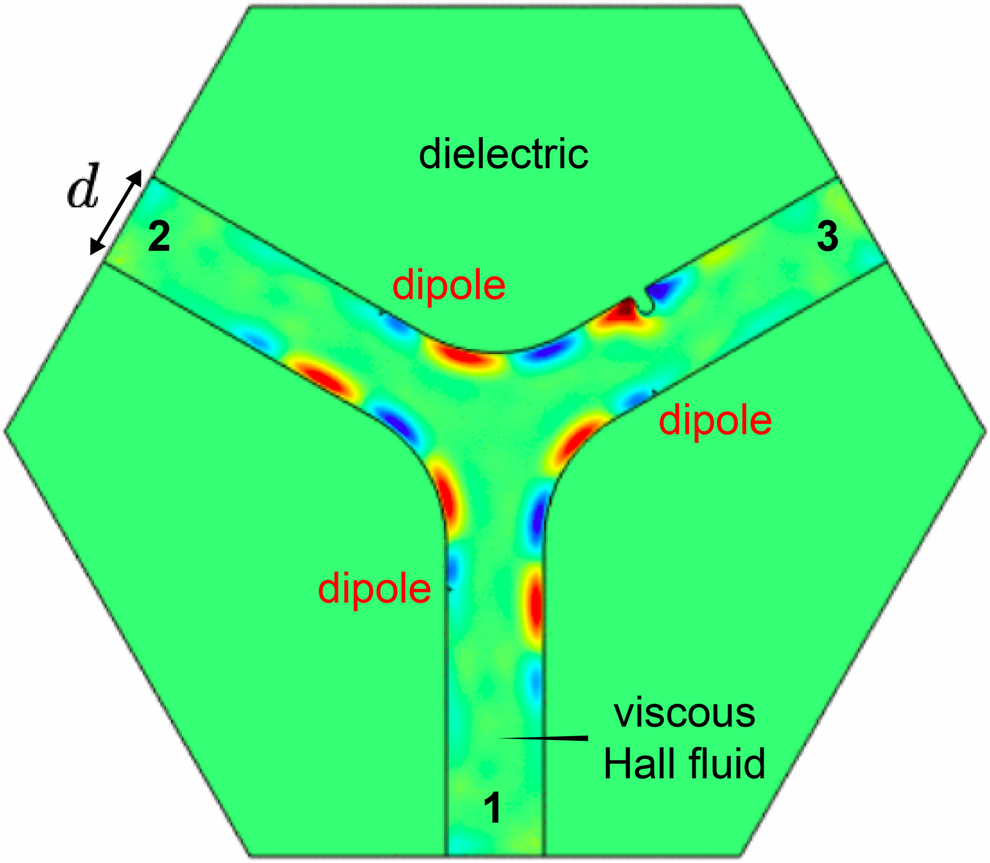

Supplementary Fig. 2: **Geometry of a 3-port ultra-subwavelength topological circulator.** The port width is $d = 274$ nm. The pathways are designed with nontrivial viscous Hall fluid $N = 2$ and surrounded by dielectric. The signals are generated by dipole sources placed at the entry ports which oscillate in the band gap $\omega/4\pi = \omega_c/4\pi = 2.26$ THz. Signals are routed from $1 \to 2 \to 3 \to 1$ with no back-scattering. A defect at port 3 is navigated with impunity.

[3] I. Torre, A. Tomadin, A. K. Geim, and M. Polini, Phys. Rev. B **92**, 165433 (2015), URL `https://link.aps.org/doi/10.1103/PhysRevB.92.165433`.

[4] P. S. Alekseev, Phys. Rev. Lett. **117**, 166601 (2016), URL `https://link.aps.org/doi/10.1103/PhysRevLett.117.166601`.

[5] F. M. D. Pellegrino, I. Torre, A. K. Geim, and M. Polini, Phys. Rev. B **94**, 155414 (2016), URL `https://link.aps.org/doi/10.1103/PhysRevB.94.155414`.

[6] E. Tiras, S. Ardali, T. Tiras, E. Arslan, S. Cakmakyapan, O. Kazar, J. Hassan, E. Janzén, and E. Ozbay, Journal of Applied Physics **113**, 043708 (2013), URL `https://doi.org/10.1063/1.4789385`.

[7] M. Müller, J. Schmalian, and L. Fritz, Phys. Rev. Lett. **103**, 025301 (2009), URL `https://link.aps.org/doi/10.1103/PhysRevLett.103.025301`.

[8] M. Mendoza, H. J. Herrmann, and S. Succi, Scientific Reports **3**, 1052 (2013), ISSN 2045-2322, URL `https://doi.org/10.1038/srep01052`.

[9] M. Sherafati, A. Principi, and G. Vignale, Phys. Rev. B **94**, 125427 (2016), URL `https://link.aps.org/doi/10.1103/PhysRevB.94.125427`.

[10] F. M. D. Pellegrino, I. Torre, and M. Polini, Phys. Rev. B **96**, 195401 (2017), URL `https://link.aps.org/doi/10.1103/`

PhysRevB.96.195401.

[11] A. Principi, G. Vignale, M. Carrega, and M. Polini, Phys. Rev. B **93**, 125410 (2016), URL https://link.aps.org/doi/10.1103/PhysRevB.93.125410.

[12] C. Hoyos and D. T. Son, Phys. Rev. Lett. **108**, 066805 (2012), URL https://link.aps.org/doi/10.1103/PhysRevLett.108.066805.

[13] T. L. Hughes, R. G. Leigh, and O. Parrikar, Phys. Rev. D **88**, 025040 (2013), URL https://link.aps.org/doi/10.1103/PhysRevD.88.025040.

[14] T. Scaffidi, N. Nandi, B. Schmidt, A. P. Mackenzie, and J. E. Moore, Phys. Rev. Lett. **118**, 226601 (2017), URL https://link.aps.org/doi/10.1103/PhysRevLett.118.226601.

[15] A. I. Berdyugin, S. G. Xu, F. M. D. Pellegrino, R. Krishna Kumar, A. Principi, I. Torre, M. Ben Shalom, T. Taniguchi, K. Watanabe, I. V. Grigorieva, et al., Science **364**, 162 (2019), ISSN 0036-8075, URL https://science.sciencemag.org/content/364/6436/162.

[16] J. E. Avron, R. Seiler, and P. G. Zograf, Phys. Rev. Lett. **75**, 697 (1995), URL https://link.aps.org/doi/10.1103/PhysRevLett.75.697.

[17] J. E. Avron, Journal of Statistical Physics **92**, 543 (1998), ISSN 1572-9613, URL https://doi.org/10.1023/A:1023084404080.

[18] D. Banerjee, A. Souslov, A. G. Abanov, and V. Vitelli, Nature Communications **8**, 1573 (2017), ISSN 2041-1723, URL https://doi.org/10.1038/s41467-017-01378-7.

[19] S. Ganeshan and A. G. Abanov, Phys. Rev. Fluids **2**, 094101 (2017), URL https://link.aps.org/doi/10.1103/PhysRevFluids.2.094101.

[20] G. Giuliani, G. Vignale, and C. U. Press, *Quantum Theory of the Electron Liquid*, Masters Series in Physics and Astronomy (Cambridge University Press, 2005), ISBN 9780521821124, URL https://books.google.com/books?id=kFkIKRfgUpsC.

[21] A. L. Fetter, Annals of Physics **81**, 367 (1973), ISSN 0003-4916, URL http://www.sciencedirect.com/science/article/pii/0003491673901619.

[22] E. B. Kolomeisky and J. P. Straley, Phys. Rev. B **96**, 165116 (2017), URL https://link.aps.org/doi/10.1103/PhysRevB.96.165116.

[23] G. Volovik, *The Universe in a Helium Droplet*, International Series of Monographs on Physics (OUP Oxford, 2009), ISBN 9780199564842, URL https://books.google.com/books?id=6uj76kFJOHEC.

[24] V. Gurarie, Phys. Rev. B **83**, 085426 (2011), URL https://link.aps.org/doi/10.1103/PhysRevB.83.085426.

[25] M. R. Hirsbrunner, T. M. Philip, and M. J. Gilbert, Phys. Rev. B **100**, 081104 (2019), URL https://link.aps.org/doi/10.1103/PhysRevB.100.081104.

[26] Y. Zhou and J. Liu, Journal of Statistical Mechanics: Theory and Experiment **2020**, 033101 (2020), URL https://doi.org/10.1088%2F1742-5468%2Fab74cc.

[27] R. Cohen and M. Goldstein, Phys. Rev. B **98**, 235103 (2018), URL https://link.aps.org/doi/10.1103/PhysRevB.98.235103.

[28] A. L. Fetter, Phys. Rev. B **32**, 7676 (1985), URL https://link.aps.org/doi/10.1103/PhysRevB.32.7676.

[29] V. A. Volkov and S. A. Mikhailov, Zhurnal Eksperimentalnoi i Teoreticheskoi Fiziki **94**, 217 (1988).
