# Supplementary material for: Optical N-invariant of graphene’s topological viscous Hall fluid
Source: Nat Commun. 2021 Aug 5;12:4729. doi: 10.1038/s41467-021-25097-2 (PMC8342470; doi:10.1038/s41467-021-25097-2)
Supplement: Supplementary file 3 — Description of Additional Supplementary Files [file 41467_2021_25097_MOESM3_ESM.pdf]

Description of Additional Supplementary Files

Supplementary Movie 1:

Time lapse of a topological edge magnetoplasmon propagating withzero backscattering along the boundary of a circulator.
