# Supplementary figures and images for: Optical N-invariant of graphene’s topological viscous Hall fluid
Source: Nat Commun. 2021 Aug 5;12:4729. doi: 10.1038/s41467-021-25097-2 (PMC8342470; doi:10.1038/s41467-021-25097-2)

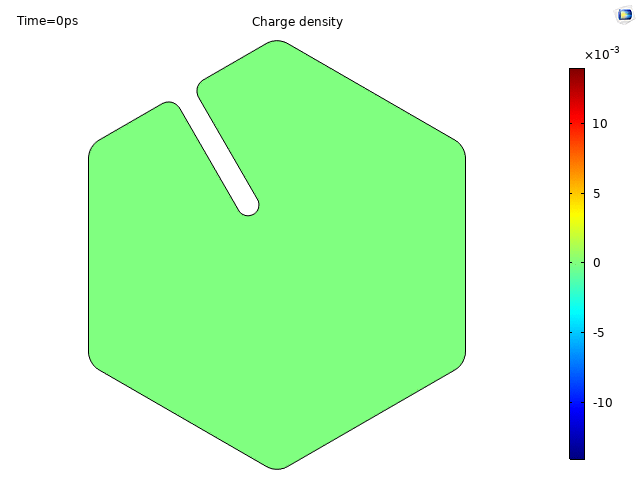

Supplement: Supplementary file 4 — Supplementary Movie 1 [file 41467_2021_25097_MOESM4_ESM.gif]
